# Alk^Tango^ reveals a role for Jeb/Alk signaling in the *Drosophila* heart

**DOI:** 10.1186/s12964-025-02150-x

**Published:** 2025-05-17

**Authors:** G Wolfstetter, T Masudi, E Uçkun, J Y Zhu, M Yi, V Anthonydhason, J Guan, H Sonnenberg, Z Han, R H Palmer

**Affiliations:** 1 Department of Medical Biochemistry and Cell Biology, Institute of Biomedicine at the Sahlgrenska Academy, University of Gothenburg, 41390 Gothenburg, Sweden; 2 Center for Precision Disease Modeling, Department of Medicine, University of Maryland School of Medicine, Baltimore, MD 21201 USA; 3 Division of Endocrinology, Diabetes and Nutrition, Department of Medicine, University of Maryland School of Medicine, Baltimore, MD 21201 USA; 4 School of Biomedical Sciences and Pharmacy, College of Health, Medicine and Wellbeing, The University of Newcastle, Callaghan, NSW 2308 Australia

**Keywords:** Tango RTK activity assay, CRISPR/Cas9, Jelly belly, Wing hearts, Cardiomyocyte, Cardiac arrythmia

## Abstract

Anaplastic lymphoma kinase (Alk) signaling is important in a variety of biological contexts such as cell type specification, regulation of metabolic and endocrine programs, behavior, and cancer. In this work, we generated a Tango GPCR assay-based, dimerization-sensitive Alk activity reporter (Alk^Tango^) and followed receptor activation throughout *Drosophila* development. Alk^Tango^ reports Alk activation in embryonic and larval tissues previously linked to Alk signaling. Remarkably, Alk^Tango^ was active in the heart of *Drosophila* larvae and adult flies. We show that cardiomyocytes express Alk from late embryonic stages to adulthood, while *jeb* expression in pericardial cells coincided with Alk^Tango^ activity. Perturbation of cardiac Alk signaling leads to decreased adult survival as well as lower fitness and increased lethality in response to heat stress. In keeping with a role for Alk, heart measurements reveal arrythmia and irregular muscle contraction upon ligand stimulation. Finally, activation of cardiac Alk signaling induces hyperplasia in the accessory wing hearts of adult flies.

**Supplementary Information:**

The online version contains supplementary material available at 10.1186/s12964-025-02150-x.

## Significance statement

Anaplastic lymphoma kinase (Alk) signaling drives highly regulated processes such as cell specification in the fruitfly to uncontrolled cellular proliferation in human cancer. Using a novel Alk activity reporter, Alk^Tango^, we have investigated endogenous Alk signaling dynamics during *Drosophila* development. Notably Alk^Tango^ identified Alk signaling events in cardiac tissues. This previously undocumented Alk signaling activity is supported by Alk expression in cardiomyocytes and Jeb expression in pericardial cells. Disrupting cardiac Alk signaling compromised adult fly survival and fitness. Moreover, Jeb-driven Alk activation induced arrhythmia and cardiac muscle irregularities and accessory wing heart hyperplasia. This study underscores the dynamic nature of Alk signaling during development, identifying a novel role in heart function in *Drosophila*, offering insights potentially translatable to human pathology.

## Introduction

Development is a dynamic, spatio-temporally controlled process which continuously integrates information from multiple signaling pathways. Receptor tyrosine kinases (RTKs) are key contributors in many aspects of multicellular development. While RTKs are widespread in the various tissues of the developing animal, their expression is not synonymous with their signaling activity. This was elegantly illustrated in the MAP kinase in situ activation atlas revealing spatio-temporal RTK activity during embryonic and larval stages of *Drosophila* development [1]. While this experimental approach highlights the difference between RTK expression and downstream activation, it requires further information from functional studies to assign MAP kinase activity to a specific receptor. Several RTK-activity reporter systems including fluorophore and FRET-based biosensors [2, 3] as well as orthogonal tags [4] have been developed. However, low receptor specificity, extensive engineering and weak readout signals make them less suitable to identify previously unknown functions of receptors with spatio-temporally complex expression profiles.

Anaplastic lymphoma kinase (Alk) is an evolutionary conserved RTK of the insulin receptor family. The activating ligands for Alk are Jelly belly (Jeb) in *Drosophila* and Hen-1 in *C. elegans* while vertebrate ALK and its paralog LTK are activated by ALKAL proteins which are phylogenetically unrelated to the invertebrate ligands [5–13]*.* Initial studies revealed essential roles for *Alk* signaling in visceral muscle founder cell specification in the fly [6, 8] and the sensory control of dauer formation in *C. elegans* [11] while aberrant ALK signaling in humans was identified as oncogenic driver and primarily studied in the context of anaplastic large cell lymphoma, neuroblastoma, and non-small cell lung-cancer [14–16]. Later studies in different model systems locate the main, evolutionary conserved roles for Alk signaling in the central and peripheral nervous system: Alk is broadly expressed in neurons of the developing nervous system and can be found at lower levels in the adult brain [17–23]. Alk signaling has been linked to neuronal growth and survival [23–26], and acts on various aspects of neuronal differentiation such as polarity determination**,** axonal targeting, synaptogenesis, and dendritic spine formation [27–30]. This might explain the vast diversity of Alk-affected traits including longevity, regulation of body growth, and sleep in *Drosophila* [18, 31–33], learning, memory formation and behavior in *C. elegans*, flies, and mice [17, 18, 34, 35], alcohol dependence in flies, mice and human [36, 37] as well as neuronal regulation of metabolic and endocrine programs in flies, honeybees, mice, and human [32, 36, 38–42]. Notably, in many of these processes, Alk signaling appears to serve as a hub between the nutritional and sensory environment and the internal regulation of metabolic and behavioral programs.

The *Drosophila* heart or dorsal vessel is a tube-shaped organ that pumps the hemolymph, the insect’s functional equivalent of blood and interstitial fluid. The heart develops from bilateral primordia in the dorsal tip of the mesoderm that subsequently migrate towards the midline and fuse into the cardiac tube. The cardiac tube consists of an inner row of contractile Tinman-positive cardiomyocytes and Seven-up positive cells, the latter representing presumptive and functional inflow tracts (ostia). An outer layer of irregularly arranged nephrocyte-type cells, the pericardial cells, flanks the muscular tube. The heart tube is embedded in a pericardin-rich extracellular matrix and attached to the body wall by alary muscles. The larval heart comprises three domains: the anterior aorta, which is flanked by the hematopoietic lymph gland, the posterior aorta that rebuilds most of the adult heart during metamorphosis, and the posterior heart chamber (also referred to as heart proper). A single cardiac valve connects aorta and heart proper. Because many aspects of dorsal-vessel formation in flies are highly reminiscent of vertebrate heart development, *Drosophila* has become a simple and accessible model to study cardiac disease [43–46].

In addition to the dorsal vessel, insects possess autonomous accessory pulsatory organs that ensure hemolymph flow into body appendages. Previous elegant studies in *Drosophila* identified a pair of sickle-shaped, bilateral, pulsatory organs in the scutellum that are derived from the cardiac mesoderm [47]. In addition to their function in physiological homeostasis, these so-called wing hearts play an essential role in wing maturation removing remnants of delaminated epithelial cells from the inner surface of the unfolded wing [48].

In this work, we developed an Alk activity reporter based on the Tango GPCR assay [49], which detects Alk activation in tissues with previously described Alk function. Unexpectedly, we also detected Alk activity in cardiomyocytes of the larval and adult dorsal vessel. Further analysis revealed cardiac Alk expression from late-stage embryos to adult flies while adjacent pericardial cells expressed Jeb. Genetic manipulation of cardiac Alk signaling revealed heartbeat changes, and aberrant marker expression in the locally specified cardiomyocytes of ostia and the larval valve. In addition, stimulation of Alk signaling induced hyperplastic muscle growth in wing hearts. Taken together, our data identify a previously unnoticed role for Alk RTK signaling in cardiac tissues.

## Results

### Alk^Tango^ monitors dimerization dependent Alk activation

**Fig. 1:**
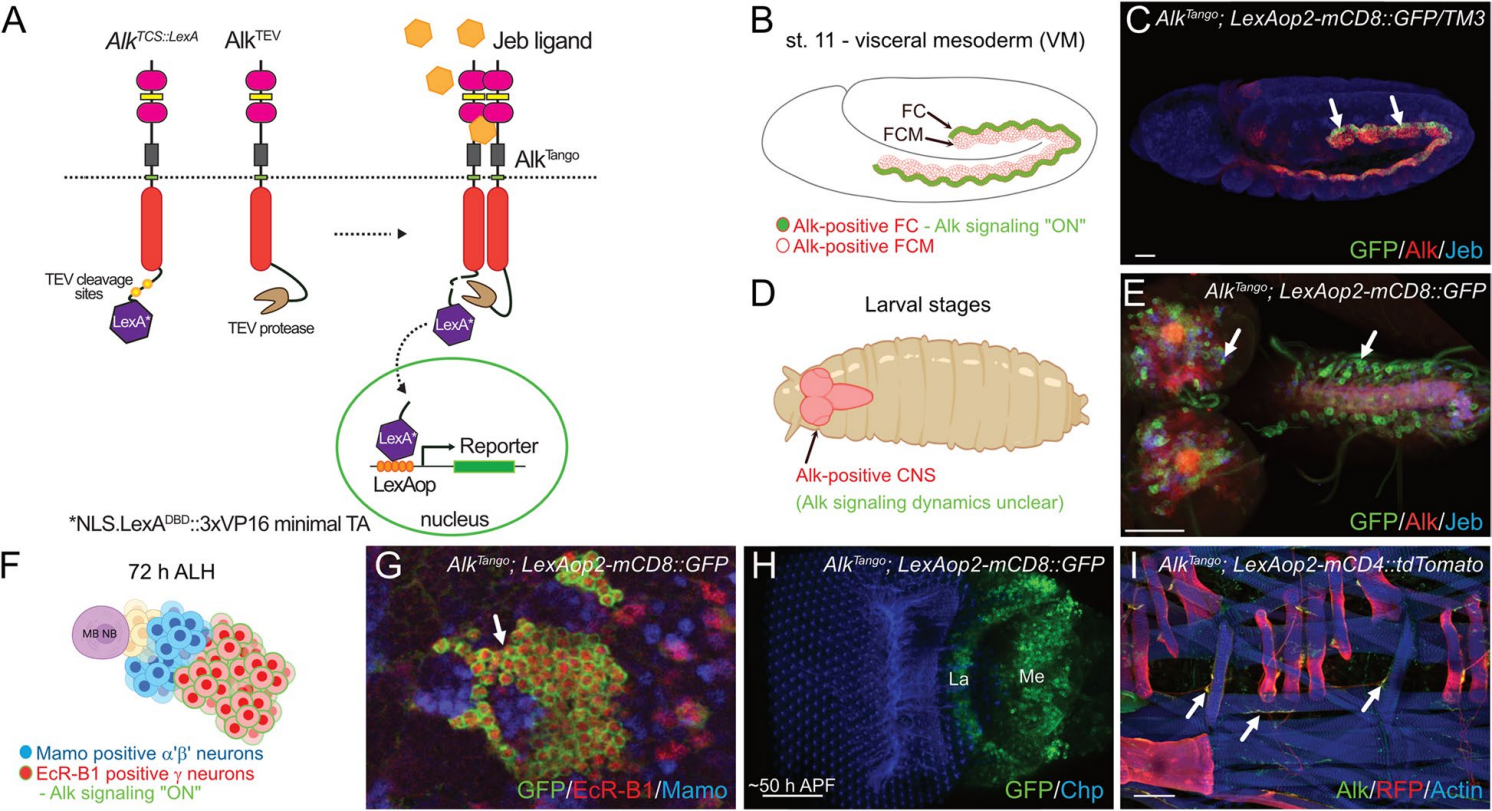
*Alk*^*Tango*^ reports Alk activation. **A** Schematic outline of the Alk^Tango^ system, comprising the *Alk*^*TCS::LexA*^ and *Alk*^*TEV*^ CRISPR modified *Alk* alleles, as well as the *LexAop2-reporter*. In brief: Alk^TCS::LexA^ and Alk^TEV^ dimerize in the presence of Alk ligand Jeb, resulting in proteolytic cleavage of an NLS.LexA^DBD^::3xVP16 minimal TA chimera (LexA*) which initiates reporter gene expression. **B** Schematic outline of Alk^Tango^ activity (depicted in green), indicating Alk activation in visceral mesoderm founder cells (arrows) of *Alk*^*Tango*^*; LexAop2-mCD8::GFP* embryos. Alk protein expression depicted in red. **C** Alk^Tango^ activity (GFP, green), indicating Alk activation in visceral mesoderm founder cells (arrows) of *Alk*^*Tango*^*; LexAop2-mCD8::GFP* embryos. Anti-Alk appears in red, anti-Jeb in blue. **D** Schematic outline of Alk expression in the larval CNS, where Alk signaling dynamics are unclear. Alk protein expression depicted in red. **E** Antibody staining revealing Alk^Tango^ activity (GFP, green) in the larval CNS of *Alk*^*Tango*^*; LexAop2-mCD8::GFP* animals. Anti-Alk appears in red, anti-Jeb in blue. **F** Schematic outline of *Alk*^*Tango*^ activation (depicted in green) in EcR-B1-positive MB γ-neurons of a third instar larva. EcR-B1 in red, mamo labels MB α’β’-neurons in blue. **G** *Alk*^*Tango*^ activation (GFP, green) in EcR-B1-positive MB γ-neurons of a third instar larva. EcR-B1 in red, mamo labels MB α’β’-neurons in blue. **H** Alk^Tango^ activity (GFP, green) in lamina and medulla neurons of a pupal optic lobe ~50 h APF. **I** Alk^Tango^ activation (RFP, red) in somatic muscles and motoneuron axons (arrows) of a third instar *Alk*^*Tango*^*; LexAop2-mCD4::tdTomato* larva. Anti-Alk appears in green, muscles stained with phalloidin (blue). Scale bars are: 20 µm in C, 50 µm in E, H, and 100 µm in I. Schematics in B, D and F created with BioRender.com (2023)

The *Drosophila* Alk receptor can be activated in exogenous models, such as the rat pheochromocytoma PC12 cell model, by recombinant Jeb ligand, leading to activation of downstream signaling and neuronal differentiation (Supplementary Fig. S1). However, following the dynamics of Alk activation in an intact organism has not been previously possible. To follow spatio-temporal Alk receptor activation in vivo*,* we adapted the G-protein coupled receptor (GPCR) Tango system, in which receptor dimerization results in TEV-protease-cleavage and nuclear localization of a transcription factor followed by reporter gene activation [49]. To do this, we generated two new *Alk* alleles using CRISPR/Cas9 mediated Homology Directed Repair (HDR) (Fig. 1A, Supplementary file 1): (1) *Alk*^*TCS::LexA*^, which encodes endogenous Alk c-terminally fused to two tobacco etch virus (TEV-)protease cleavage sites (TCS) followed by a nuclear localization signal (NLS), the LexA DNA binding domain (DBD) fused to a 3xVP16 minimal trans-activation domain (3xVP16minimalTA) [50], and (2) *Alk*^*TEV*^, which is a c-terminal fusion of endogenous Alk with a synthetic TEV-protease variant.

We crossed *Alk*^*TCS::LexA*^ and *Alk*^*TEV*^ flies in the background of *LexAop2-*regulated fluorescent protein reporters and analyzed F1 animals that exhibited reporter activity (hereafter referred to as Alk^Tango^). In agreement with our previous knowledge of Alk activation in the embryonic visceral mesoderm (Fig. 1B), we observed robust Alk^Tango^ signals in the muscle founder cell row of this Alk expressing tissue (Fig. 1C**, **arrows). Alk signaling also plays important roles in a variety of neuronally regulated processes (Fig. 1D). In line with this, we observed Alk^Tango^ activity in a subset of Alk expressing neurons in the brain lobes, as well as in the neuropil and VNC neurons (Fig. 1E). Alk is expressed in the mushroom body lineages [25] and Alk^Tango^ activity in the central nervous system (CNS) was found in the EcR-B1-positive mushroom body γ-lineage but not the Mamo-expressing α´β´-neurons (Fig. 1F-G) which agrees with our recent observation that aberrant Alk signaling perturbs neuronal fate of the mushroom body γ-lineage [25]**.** An additional site of Alk expression in the brain is in the medulla of the optic lobe [27], where it has been reported to play a role in retinal axon targeting [27]. We observed *Alk*^*Tango*^ activity in the medulla and lamina plexus of the optic lobe during pupal development (Fig. 1H). In addition, we detected weak Alk^Tango^ activity in body wall muscles (Fig. 1I) which is in line with an earlier described role of Alk signaling in the postsynaptic density of larval motor neurons [30]. Notably, axons of larval motor neurons also exhibited Alk^Tango^ activity (Fig. 1I**, **arrows) suggesting a previously unknown upstream function for Alk signaling. Taken together, these observations suggest that the Alk^Tango^ reporter system reveals Alk dimerization in a ligand-dependent manner and therefore most likely reflects Alk activation in vivo.

### Alk^Tango^ reveals Alk signaling activity in the dorsal vessel of larvae and adult flies

**Fig. 2:**
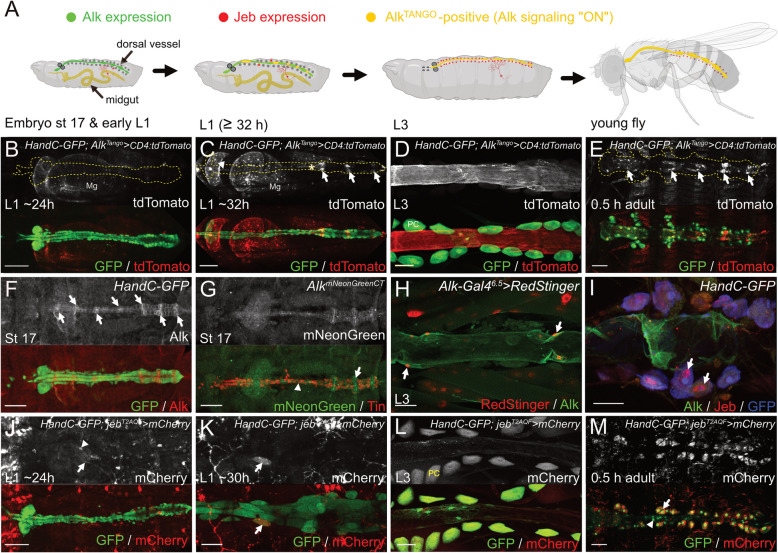
Alk and Jeb are expressed in adjacent cell types in the dorsal vessel. **A** Schematic created with BioRender.com (2023) summarizing cardiac Alk expression, *jeb*^*T2A*^ reporter expression in surrounding tissues as well as cardiac Alk^Tango^ reporter activity in different developmental stages. Green indicates Alk expression with no or low Alk^Tango^ reporter activity, Jeb expressing tissues are marked in red, Alk^Tango^ reporter activity is shown in yellow, HandC-GFP positive lymph gland and PCs are denoted in dark grey. **B** Alk^Tango^ reporter activity (white, red in merge) in a freshly hatched L1 larva. The *HandC-GFP* reporter (green) was used to label the dorsal vessel (encircled area in b/w images), Tango associated tdTomato expression is only visible in the larval midgut (Mg). **C** Six hours after hatching, Alk^Tango^ reporter activity (white, red in merge) is now visible in aorta (arrowhead), ostia (arrows), and the valve region (asterisk) of the HandC-GFP labeled (green) dorsal vessel. **D** Antibody staining against HandC-GFP (green) and tdTomato (anti RFP antibody) reveals persistent Alk^Tango^ reporter activity (white, red in merge) in cardiomyocytes of a wandering third instar larva. PC = pericardial cell. **E** Alk^Tango^ reporter activity (white, red in merge) in the adult heart (encircled area in b/w image) ~30 min after emergence. Heart cells are labeled by the HandC-GFP reporter, the highest Alk^Tango^ signals appear in ostia (arrows). **F** Anti-Alk antibody staining (white, red in merge) in cardiomyocytes of the HandC-GFP-positive (green) dorsal vessel of a late-stage *Drosophila* embryo. Arrows label presumptive ostia. **G** Anti-Tin (red) and mNeonGreen (white, green in merge) antibody staining reveals Alk^mNeonGreenCT^ expression in Tin-positive cardiomyocytes (arrowhead) and Tin-negative ostia (arrow) of a late-stage *Drosophila* embryo. **H** Antibody staining of the dorsal vessel of a wandering L3 larva expressing *UAS-RedStinger* (red) under control of *Alk*^*P6.5*^*-Gal4*. Anti Alk antibody staining appears in green; arrows indicate cardiomyocyte nuclei. **I** Alk expression in the adult heart of a *HandC-GFP* fly stained with anti-Alk (green), anti-Jeb (red), and anti-GFP (blue). Arrows indicate Jeb antibody staining in PCs. **J** *Jeb*^*T2A-QF*^ driven *QUAS-mCherry* expression (white, red in merge) in a freshly hatched L1 larva. An arrow marks dendritic branches of a *Jeb*^*T2A*^-positive ddaC neuron, arrowhead labels an unspecified *Jeb*^*T2A*^-positive cell close to the heart. **K** Six hours afterhatching, *Jeb*^*T2A-QF*^ driven *QUAS-mCherry* expression (white, red in merge)can be detected in single pericardial cells (arrow) of the dorsal vessel (labeled green by HandC-GFP). **L** Antibody staining against mCherry (anti-RFP antibody) and *HandC*-driven GFP reveals Jeb reporter activity in pericardial cells (PC) of a third instar larva. **M** Jeb reporter activity appears in pericardial cells (arrow) but not cardiomyocytes (arrowhead) of the adult heart ~30 min after emergence. Scale bars are 10 µm in J; 20 µm in E and F; 50 µm in B, C, G, H, I, and K; 100 µm in D, and L

In addition to our observations of Alk^Tango^ reporter activity in previously reported Alk-driven processes (Fig. 1), we detected Alk^Tango^ activity in the larval dorsal vessel (Fig. 2A). We therefore followed Alk^Tango^ activation in the background of the *HandC-GFP* reporter which is expressed in the embryonic lymph gland and dorsal vessel [47, 51]. In contrast to Alk^Tango^ signals in the midgut muscle layer (Mg), we did not detect Alk^Tango^ activity in the HandC-GFP-labelled dorsal vessel of freshly hatched L1 larvae (Fig. 2B). However, at later time points (~ 6 h after hatching), cardiac Alk^Tango^ activity was visible (Fig. 2C). Interestingly, we observed strong Alk^Tango^ signals in the ostia of the heart proper (Fig. 2C, arrows), but also the valve region (Fig. 2C**, **asterisk) as well as cardiomyocytes of the aorta exhibited reporter activity (Fig. 2C**, **arrowhead). Dissection and staining of dorsal vessels from wandering third instar larvae revealed robust Alk^Tango^ activation in cardiomyocytes of aorta and heart but not in adjacent, HandC-GFP-positive, pericardial cells (Fig. 2D**, **PC). Furthermore, Alk^Tango^ signals also appeared in adult cardiomyocytes ~ 0.5 h after emergence (Fig. 2E**)**. In line with our observations at earlier developmental stages, Alk^Tango^ signals were strongest in the ostia (Fig. 2E**, **arrows) of adult flies. Together, these observations identify a previously unappreciated Alk signaling dynamic in the *Drosophila* heart that is initiated in the early larval stages, and which persists to adulthood.

### Adjacent cardiac cell types express Alk and Jeb

These findings further prompted us to investigate Alk protein expression in cardiac tissues during development. During early embryogenesis, Alk expression labels cells of the visceral mesoderm primordia but not the adjacent cardiac mesoderm (Supplementary Fig. 2A). Analyzing Alk protein expression in *HandC-GFP* embryos, we found initial weak Alk expression in the bilaterally specified heart progenitor cells that migrated towards the midline (Supplementary Fig. 2B-C). At the end of embryogenesis, we detected Alk expression in cardiomyocytes of both aorta and heart proper, with higher expression levels in presumptive ostia (Fig. 2F**, **arrows). To further validate our observations, we used CRISPR/Cas9 mediated HDR to generate a mNeonGreen-tagged *Alk* allele by inserting a codon-optimized *mNeonGreen*-encoding sequence c-terminally in frame with *Alk* (Supplementary file 1). The resulting *Alk*^*mNeonGreenCT*^ animals displayed the expected Alk expression in the embryonic visceral mesoderm and CNS and were homozygous viable and fertile, suggesting that addition of the mNeonGreen moiety did not perturb Alk signaling. In agreement with our previous findings, Tin-positive cardiomyocytes (Fig. 2G**, **arrowhead) as well as Tin-negative ostial cells expressed Alk^mNeonGreenCT^ (Fig. 2G**, **arrow). The presence of Alk in the dorsal vessel was further validated with the *AlkP*^*6.5*^ reporter line, which is expressed in the embryonic visceral mesoderm [52], as well as in the larval somatic musculature. In agreement with the Alk protein expression and Alk^Tango^ reporter activity, we observed that the *AlkP*^*6.5*^ reporter was active in cardiomyocytes of wandering third instar larvae (Fig. 2H**, **arrows). Moreover, antibody staining also revealed Alk expression in heart muscles of *HandC-GFP* third instar larvae and adult flies (Fig. 2H and I). Of note, *AlkP*^*6.5*^ reporter activity (RedStinger-positive nuclei in Fig. 2H**, **asterisks) in the larval somatic musculature together with Alk protein expression at the neuromuscular junction, reflect the known localization of Alk at the postsynaptic density [30]. Having confirmed Alk expression in the dorsal vessel from late embryonic stages, we next investigated expression of the Jelly belly (Jeb) ligand. We noticed that Jeb antibody staining frequently labelled pericardial cells flanking the adult heart (Fig. 2I**, **arrows). Pericardial cells are functional nephrocytes [53] which could imply that the anti-Jeb signals in these cells are a result of the macromolecular uptake of Jeb protein from the hemolymph. We therefore employed a T2A trojan exon insertion [54] in the endogenous Jeb locus (*jeb*^*T2A−QF*^, gift from Michael O´Connor) and followed *QUAS-mCherry* reporter expression in a *HandC-GFP* background. In agreement with our observations in the Alk^Tango^ system, we did not detect Jeb reporter activity in the dorsal vessel of freshly hatched L1 larvae (Fig. 2J) although closely associated structures like the dendrites of the peripheral ddaC neurons (Fig. 2J**, **arrows; Supplementary Fig. 2) and some unspecified cells (Fig. 2J**, **arrowhead) appeared *jeb*^*T2A−QF*^ > *QUAS-mCherry* positive. Six hours after hatching however, we observed mCherry expression in single HandC-GFP-positive pericardial cells (Fig. 2K**, **arrow). Further antibody staining of dissected dorsal vessels from *jeb*^*T2A−QF*^ > *QUAS-mCherry* third instar larvae (L3) revealed *jeb* reporter activity in all pericardial cells (Fig. 2L**, **PC). In agreement with these observations, adult pericardial cells (Fig. 2M**, **arrow) but not cardiomyocytes of the heart tube (Fig. 2M**, **arrowhead) exhibited strong *jeb*^*T2A*^ reporter activity ~ 0.5 h after emergence. Taken together, we identified Alk expression in cardiomyocytes from late embryonic stages followed by Jeb expression in the adjacent pericardial cells of L1 larvae. In addition, *Alk*^*Tango*^ activity coincided with Jeb reporter expression in adjacent cardiac cells, suggesting similarities between cardiac Alk signaling and the directional signaling mechanisms previously described in the visceral mesoderm and the optic lobe [6, 8, 27].

### Alk is not critically required for development of the embryonic dorsal vessel

**Fig. 3:**
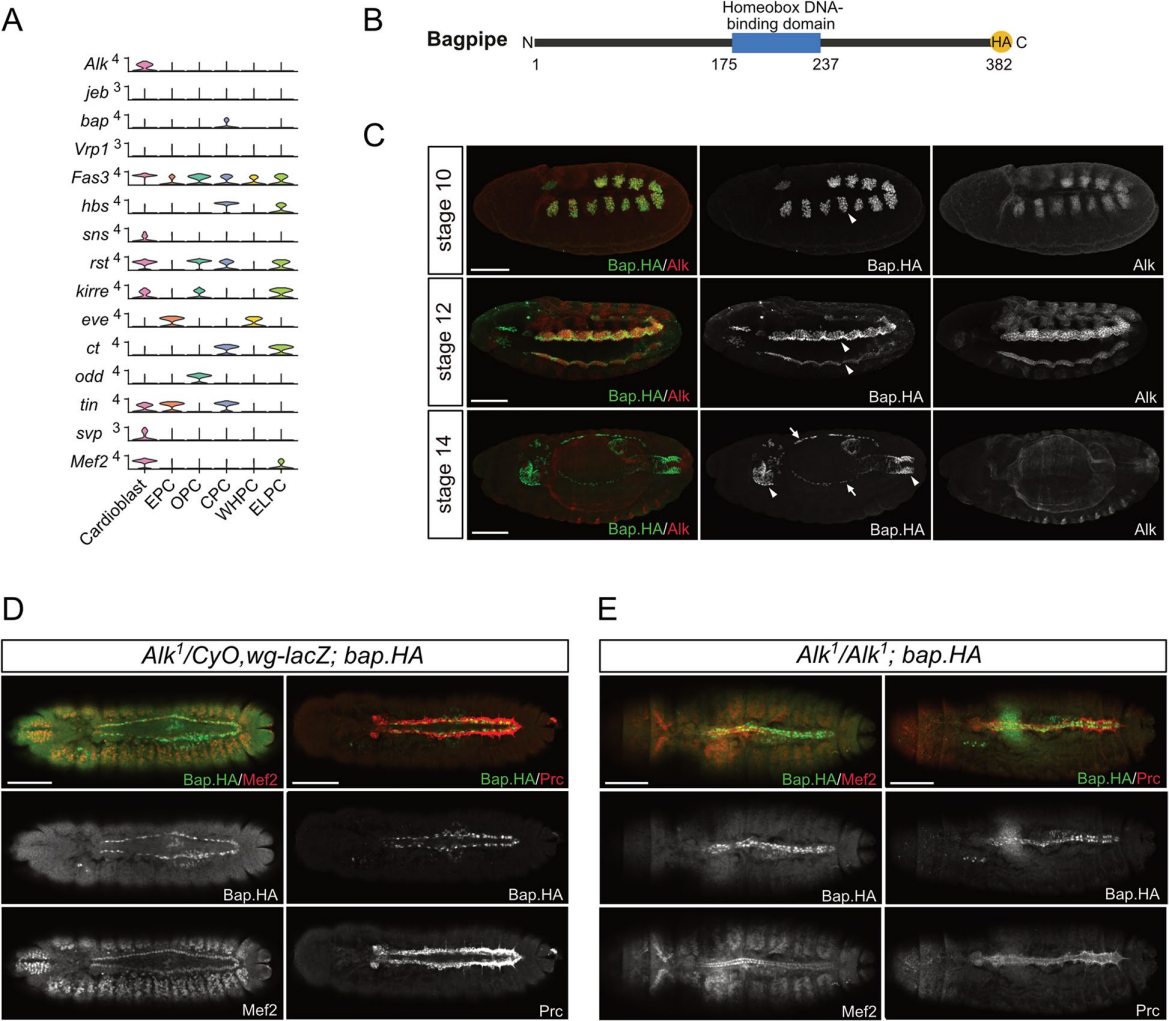
Alk mutants do not display obvious defects in specification of the embryonic heart. **A** Stacked violin plot illustrating the expression levels of genes associated with VM fusion downstream of Alk. The genes shown are *Alk, jeb, bap, Vrp1, Fas3, hbs, sns, rst, kirre, eve, ct, odd, tin, svp* and *Mef2*. Each column represents a cell cluster identified within the cardiogenic progenitor population, and the stacked violins depict the distribution of expression for each gene across clusters. Data was derived from the single-cell RNA sequencing dataset (GSE168774) and the plot was generated using the scCustomize R package. **B** Schematic representation of the CRISPR/Cas9-mediated C-terminal HA tag knock-in at the endogenous *bap* gene locus, creating the *bap*^*HA*^ allele. **C** *bap*^*HA*^ embryos stained for HA (green) and Alk (red). Bap^HA^ is expressed in the VM at stages 10-12 where it exhibits strong nuclear localization in founder cells at stage 12 (arrowheads). Expression is observed in both the foregut and hindgut at stage 14 (arrowheads). Bap^HA^ is also present in early cardiac precursor cells at stage 14 (arrows). **D-E** Stage 15–16 embryos stained for Bap^HA^ (green) and the heart markers Mef2 and Prc (red). In *Alk*^*1*^ heterozygote controls, Bap is expressed in pericardial cells (D). In *Alk*^*1*^ mutants, the midgut development is affected, but the heart structure and the expression of heart markers, including Bap, Mef2, and Prc, remain unaffected (E)

Recent single cell sequencing efforts have provided extensive information regarding tissue specific expression in the *Drosophila* embryo. We re-analyzed a previously published single-cell sequencing dataset on the developing embryonic cardiac system from Huang et al., who also noted Alk expression in the heart [55], focusing on components of the Alk signaling pathway as well as molecules known to be required in embryonic visceral mesoderm development. Our analyses confirmed robust *Alk* mRNA expression in cardioblasts (Fig. 3A). We additionally investigated the expression of the other components of the Alk signaling pathway and visceral mesoderm development, identifying expression of the *hbs*, *sns*, *rst* and *kirre* immunoglobulin heterophilic adhesion molecules, which are important for myoblast fusion, in cardiac cell populations. We also detected expression of the *bagpipe* (*bap*) homeodomain transcription factor [56], in pericardial cells (Fig. 3A) [57, 58]. To confirm the expression of *bap* in the embryonic heart, we generated a *bap*^*HA*^ allele using CRISPR/Cas9-mediated genome editing to induce HDR in the *Drosophila bap* locus (Fig. 3B-C). Anti-HA antibody staining results indeed confirmed Bap^HA^ expression in pericardial cells of the developing embryonic dorsal vessel as well as in the previously reported embryonic visceral mesoderm (Fig. 3C-D; Supplementary Fig. 3). The robust *Alk*^*Tango*^ activity observed in the dorsal vessel, together with the expression of Jeb and Alk, together with other components involved in visceral mesoderm cell specification and fusion suggested a previously unidentified cardiac function for Alk. We therefore investigated dorsal vessel during development in *Alk*^*1*^ loss of function mutants but were unable to detect any significant defects in expression of either Bap^HA^, Mef2 or Pericardin (Prc) in *Alk*^*1*^ mutant embryos when compared with controls (Fig. 3D-E). Together, these findings suggest that while Alk and other key regulators of Alk-regulated visceral mesoderm developmental processes are expressed in the embryonic heart, Alk is not critically required for specification of the dorsal vessel during embryonic development.

### Alk signaling affects later stages of heart development

**Fig. 4:**
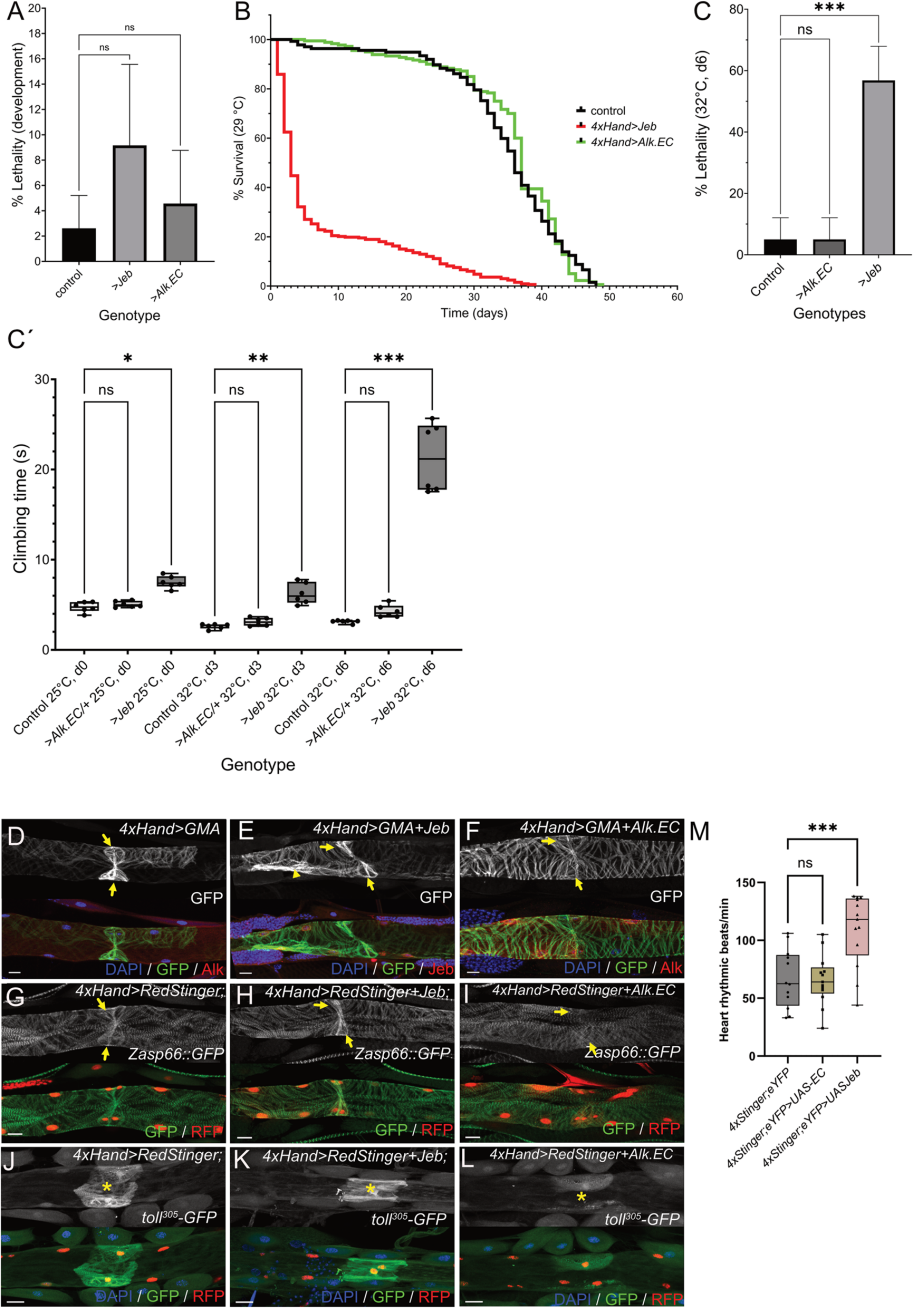
Jeb overexpression affects later heart function. **A **Column graph depicting developmental lethality (in %) of *4xHand-Gal4* control (mean = 2.612 +/- 2.594 SD), *4xHand>UAS-jeb* (mean = 9.166 +/- 6.396 SD), and *4xHand>UAS-Alk.EC *(mean = 4.569 +/- 4.201 SD). One way Analysis of Variance (ANOVA) reveals no significant (ns) difference between the genotypes. **B **Kaplan Meier curve of *4xHand-Gal4* control, *4xHand>UAS-jeb* and *4xHand>UAS-Alk.EC* flies cultured on standard diet at 29 °C. **C **Column graph depicting lethality (in %) *4xHand-Gal4* control (mean = 5 +/- 7.07 SD), *4xHand>UAS-jeb* (mean = 56.8 +/- 11.1 SD), and *4xHand>UAS-Alk.EC *(5 +/- 7.07 SD). Kruskal-Wallis test was used to reveal statistical significance (*** = *P* < 0.001, ns = non-significant). **C**´ Bee swarm box and whiskers plot depicting climbing performance after exposure to 32°C at the indicated time points. Single dots represent average values from three measurements from groups of ten flies (*n* = 60 flies for each genotype). One way Analysis of Variance (ANOVA) was used to test for statistical significance (*** = *P* < 0.001, ** = P 0.009, * = P 0.048, ns = non-significant). **D-F **Anti-GFP (green), anti-Alk or anti-Jeb (red) antibody staining of dorsal vessels from third instar *4xHand>GMA* control (D), *4xHand>GMA+Jeb* (E) and* 4xHand>GMA+Alk.EC *(F) 3^rd^ instar larvae. DAPI (blue) labels nuclei. Arrows highlight the ostial myofibrillar content. **G-I **Anti-GFP (green) and anti-RFP (red) antibody staining of dorsal vessels from third instar *4xHand>RedStinger *control (G), *4xHand>RedStinger+Jeb* (H) and *4xHand>RedStinger+Alk.EC* (I) larvae in the *Zasp66::GFP* background. Arrows highlight the ostial myofibrillar content. **J-L** Anti-GFP (green) and anti-RFP (red) antibody staining of dorsal vessels from *4xHand>RedStinger* control (J), *4xHand>RedStinger+Jeb* (K) and *4xHand>RedStinger+Alk.EC* (L) 3^rd^ instar larvae in the background of the *toll*^*305*^*-GFP* reporter. DAPI (blue) labels nuclei. Asterisks mark the position of the cardiac valve. **M** Quantification of pupal heart rates for control *4xHand-Gal4>UAS-RedStinger/+; UAS-EYFP/+*, *4xHand-Gal4>UAS-RedStinger/UAS-jeb; UAS-EYFP/+ and 4xHand-Gal4>UAS-RedStinger/+; UAS-EYFP/UAS-Alk.EC* pupae (*n* = 12 animals for each genotype). Students t-test was applied to test for statistical significance (*** = *P* < 0.001, ns = non-significant)

While we were unable to detect any embryonic heart defects in Alk mutants, the *Alk*^*Tango*^ activity in the dorsal vessel suggests a later cardiac function for Alk. We therefore genetically manipulated cardiac Alk signaling using the *4xHand-Gal4* driver in combination with either Alk-activating *UAS-jeb* or the dominant negative *UAS-Alk.EC* variant. Manipulation of cardiac Alk signaling with either *UAS-jeb* or *UAS-Alk.EC* did not affect developmental lethality (Fig. 4A), however adult flies that ectopically expressed Jeb in cardiac tissues exhibited strongly decreased survival (Fig. 4B), especially within the first four days after hatching (survival d4: control = 97.8%, *4xHand* > *jeb* = 32.13%, *4xHand* > *Alk.EC* = 100%. n > 200 animals). We did not observe a similar effect in a four-day control experiment with the combined cardiac expression of *UAS-jeb* and *UAS-Alk.EC* (survival d4: balanced siblings = 94.75%, *4xHand* > *jeb* + *ALK.EC* = 95.13%. *n* = 121 respectively 180 animals) further suggesting a specific effect of cardiac Alk activation. In addition, *4xHand* > *UAS-jeb* animals exhibited a 22% reduction in lifespan while expression of Alk.EC had no effect (Fig. 4B**)**. We next tested *4xHand* > control, *4xHand* > *UAS-jeb*, and *4xHand* > *UAS-Alk.EC* flies in a modified cardiac performance assay [59]. We exposed five-day old flies to heat stress conditions (32 °C) for six days and scored for survival. In addition, we monitored general fitness in a climbing assay at 0 days (d0), 3 days (d3) and at the end of heat exposure (d6) **(**Fig. 4C**’)**. We noted that Jeb-induced cardiac Alk signaling significantly increased lethality under heat exposure while dominant negative *UAS-Alk.EC* had no obvious effect on viability (Fig. 4C). In addition, climbing performance of *4xHand* > *UAS-jeb* animals, which was already lower at d0 compared to control and *4xHand* > *UAS-Alk.EC,* gradually decreased over the course of the experiment (Fig. 4C**’**) further indicating impaired cardiac function. We then analyzed the morphology of larval heart muscles using a *4xHand*-driven GFP-tagged moesin actin-binding domain (*UAS-GMA*, Fig. 4D-F). At the positions of the presumptive ostia in the aorta, the cardiac muscle layer exhibits increased myofibrillar content and therefore appears as sand-clock shaped structure (Fig. 4D**, **arrows). This characteristic structure was present in *4xHand* > *UAS-GMA* + *UAS-jeb* animals (Fig. 4E**, **arrows) and we observed stronger GMA-reporter signals also in adjacent parts of the cardiac muscle (Fig. 4E**, **arrowheads). In contrast, Alk.EC overexpression with *4xHand-Gal4* resulted in perturbed GMA-reporter activity throughout the larval heart (Fig. 4F**, **arrows). Because *Hand* is a known downstream target of Alk signaling in the *Drosophila* visceral mesoderm [60], altered Alk signaling could also impact on the activity of the *4xHand-Gal4* driver. We therefore analyzed cardiac muscle morphology in the background of a *Zasp66::GFP* protein trap allele (Fig. 4G-I). In agreement with our findings with *4xHand-*driven GMA, we observed increased Zasp::GFP signals at the ostia regions of *4xHand* > *UAS-jeb* animals (Fig. 4H**, **arrows) in comparison to control larvae (Fig. 4G**, **arrows), and a decrease in Zasp::GFP when Alk.EC was overexpressed with *4xHand-Gal4* (Fig. 4I**, **arrows)*.* In addition, cardiac overexpression of Alk.EC led to decreased *toll*^*305*^*-GFP* reporter activity in the cardiac valve when compared to control and *4xHand* > *UAS-jeb larvae* (Fig. 4J-L**, **asterisks).

### Alk signaling affects pupal and adult cardiac function

Taken together, this data indicates that Alk signaling alters expression of cardiac genes which could further result in physiological misfunctions that eventually affect viability and health of the animal. We next analyzed video recordings of intact pupal dorsal vessels under control conditions (*4xHand-Gal4* > *UAS-RedStinger/* + *; UAS-EYFP/* +) and upon altered Alk signaling (*4xHand-Gal4* > *UAS-RedStinger/UAS-jeb; UAS-EYFP/* + *, 4xHand-Gal4* > *UAS-RedStinger/* + *; UAS-EYFP/UAS-Alk.EC*) (Fig. 4M, Supplementary movies M1-3). In contrast to the regular heartbeat pattern of control animals, we observed cardiac arrythmia and irregular myofibrillar contractions in *4xHand-Gal4* > *UAS-RedStinger/UAS-jeb; UAS-EYFP/* + animals (Supplementary movies M1, M2) which also displayed an overall increased heart rate **(**Fig. 4M**)**. *4xHand-Gal4* > *UAS-RedStinger/UAS-Alk.EC; UAS-EYFP/* + animals did not exhibit obvious differences from control pupae (Fig. 4M, Supplementary movie M3).

**Fig. 5:**
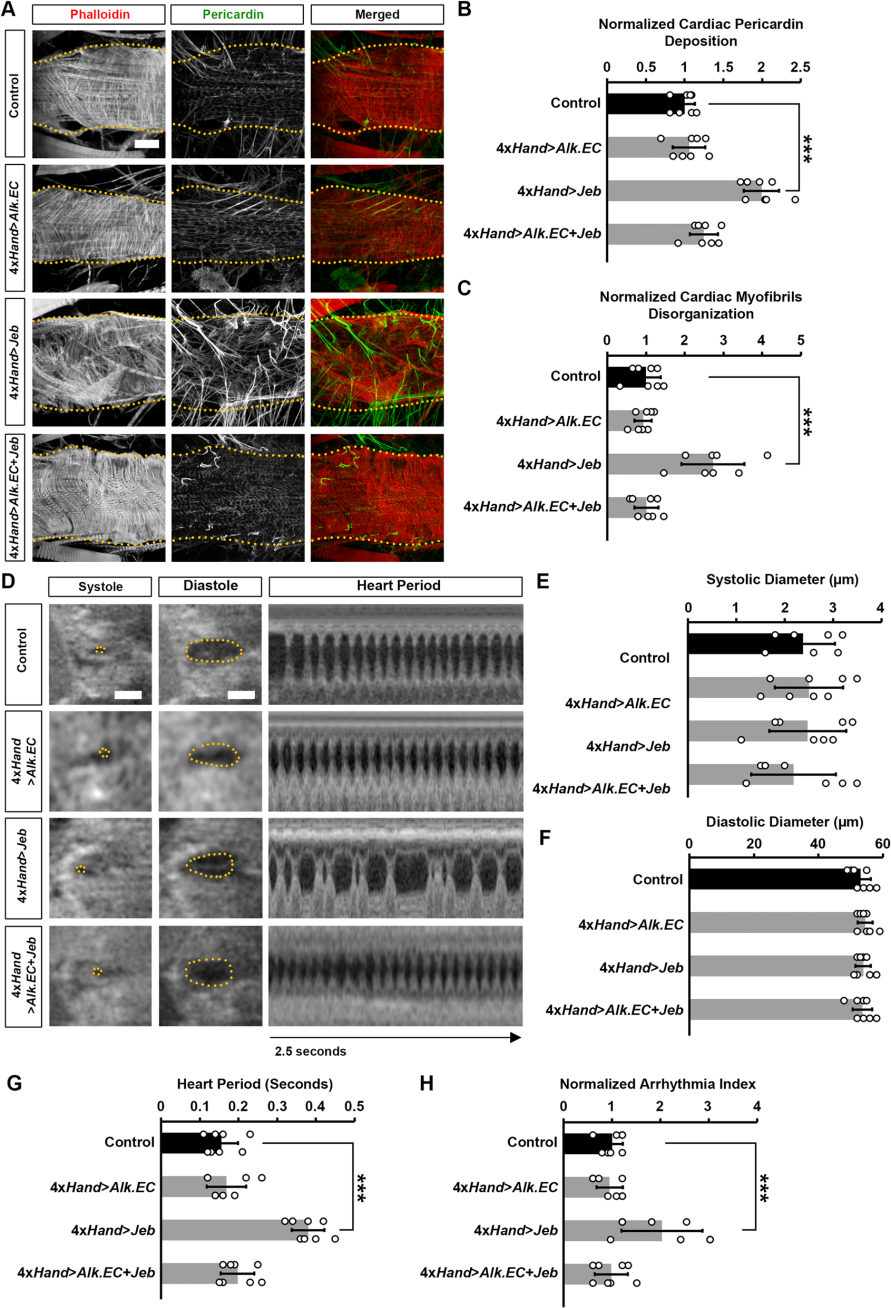
Heart structure, pericardin deposition and cardiac function in flies following heart-specific expression of Jeb and Alk.EC. **A **Adult (5-day-old females) heart phenotype induced by expression of either Jeb or dominant negative Alk.EC alone or in combination. Cardiac actin myofibers were visualized by phalloidin staining (red). Pericardin was detected by immunofluorescence (green). Dotted lines delineate the outline of the heart tube. Scale bar = 40 µm. **B **Quantification of adult heart Pericardin deposition relative to control. *n*=6 flies (5-day-old females) per genotype. **C **Quantification of cardiac myofibril disorganization relative to control. *n*=6 flies (5-day-old females) per genotype. **D** Images from *Drosophila* (4-day-old females) heartbeat videos obtained by optical coherence tomography (OCT). Representative images show changes in heart function induced by expression of either Jeb or dominant negative Alk.EC alone or in combination. Scale bar = 40 µm.** E** Quantitation of adult heart systolic diameter. *n*=10 flies (4-day-old females) per genotype. **F** Quantitation of adult heart diastolic diameter. *n*=10 flies (4-day-old females) per genotype.** G **Quantitation of heart period. *n*=10 flies (4-day-old females) per genotype (see A). Values are presented as mean along with the standard deviation (s.d). Statistical significance (*) was defined as ***P < 0.001 using Kruskal-Wallis H-test followed by a Dunn’s test. **H **Quantitation of arrythmia index (standard deviation of the heart period) relative to control. *n*=10 flies (4-day-old females) per genotype (see A). Values are presented as mean along with the standard deviation (s.d). Statistical significance (*) was defined as ***P < 0.001 using Kruskal-Wallis H-test followed by a Dunn’s test

To further examine adult fly heart structure and functional changes, we employed *4xHand-Gal4* combined with Alk-activating *UAS-jeb* or the dominant negative *UAS-Alk.EC* (Fig. 5). Hearts were stained with phalloidin to visualize the cardiac actin filaments. Ectopic expression of Jeb in the heart was associated with overall disorganization of cardiac actin filaments (Fig. 5A-C). We also observed increased deposition of Pericardin, a type IV collagen that plays a critical role in maintaining cardiac tissue integrity (Fig. 5A-B). The overabundance of Pericardin indicates a pathophysiological condition of fibrosis. We did not observe any heart structure defects as a result of Alk.EC expression.

To assess cardiac functional defects induced by expressing either Jeb or Alk.EC, we applied optical coherence tomography (OCT). The orthogonal view of the heart provides accurate and real-time measurements of the heart tube diameter and heart period. Expression of either Jeb or the dominant negative Alk.EC did not change diastolic and systolic diameters (Fig. 5D-F). However, compared to control flies, the heart period in Jeb expressing flies was significantly increased (Fig. 5G) and displayed an arrhythmia phenotype (Fig. 5D, H). Importantly, this arrhythmic phenotype was abrogated when Alk.EC was co-expressed with Jeb, indicating that Alk signaling in the heart itself is responsible for the increased heart period induced by Jeb (Fig. 5D,H; Supplementary Fig. 4).

### Increased Alk signaling induces wing heart hyperplasia

**Fig. 6:**
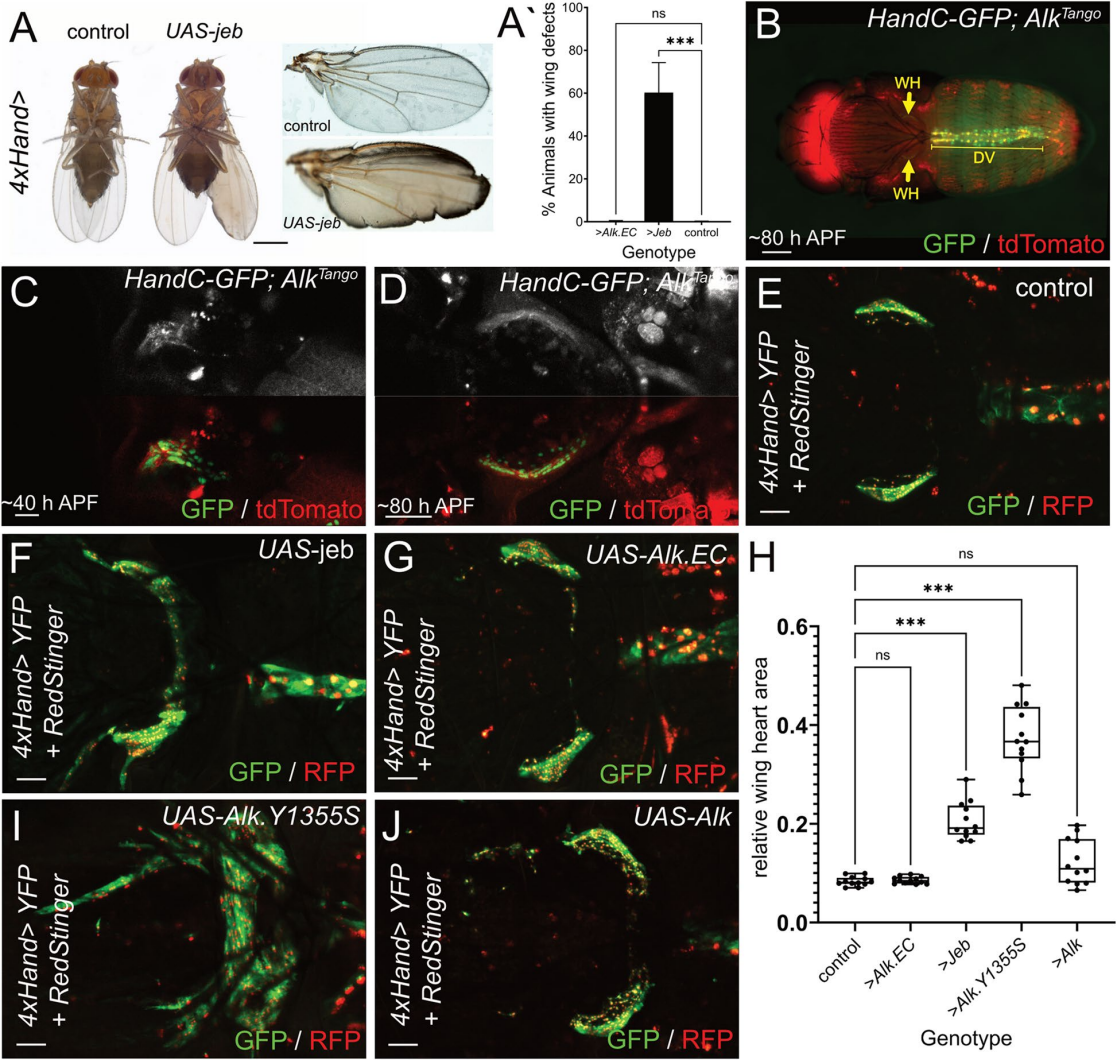
Increased Alk signaling induces wing heart hyperplasia. **A **Representative images of 1-day old control and *4xHand>UAS-jeb* flies.** A´ **Column graph depicting the percentage of animals with a wing maturation phenotype. Means and SDs are: *4xHand>* control = 0.125 +/- 0.2315, *4xHand>UAS-jeb* = 60.28 +/- 14.05, and *4xHand>UAS-Alk.EC *= 0.1875 +/- 0.3720. Kruskal-Wallis One-way analysis of variance reveals statistical significance (***= *P* < 0.001, ns = non-significant). n ≥ 700 flies per genotype were scored. **B** Localization of cardiac organs in an *HandC-GFP; Alk*^*Tango*^ (*Alk*^*TCS::LexA*^*/Alk*^*TEV*^*;* *LexAop2-CD4-tdTomato*/+) animal ~80 hours after pupa formation (APF). DV = dorsal vessel, arrows indicate the position of the wing hearts (WH). **C, D** *Alk*^*Tango*^ reporter activity in developing *HandC-GFP* labelled wing hearts at ~40 hours APF and in differentiated wing hearts at ~80 hours APF. **E-G** Wing hearts of pharate adult flies from *UAS-RedStinger/+; 4xHand-Gal4/+ ;UAS-EYFP/+* controls (E), as well as *UAS-RedStinger/+; 4xHand-Gal4/UAS-jeb; UAS-EYFP/+* (F), and *UAS-RedStinger/+; 4xHand-Gal4/+;UAS-EYFP/UAS-Alk.EC* (G) animals. **H** Bee swarm box and whiskers plot depicting relative wing heart areas from dorsal-view thorax confocal scans. Brown-Forsythe and WelchAnalysis of variance was applied to reveal statistical significance (*** *P*<0.001, ns = non-significant).** I-J** Wing hearts of pharate adult flies from *UAS-Alk.Y1355S*/*UAS-RedStinger; 4xHand-Gal4 *(I), and *UAS-Alk*/*UAS-RedStinger; 4xHand-Gal4 *(J) animals. Scale bars are 500 µm in A; 200 µm in B; 20 µm in C, and 50 µm in D-G, I, and J

When we genetically manipulated Alk signaling in the dorsal vessel we noticed that 60% of 4*xHand* > *UAS-jeb* flies displayed long term wing maturation defects. Instead of a fused wing blade, liquid accumulated between the cuticle sheets preventing tight bonding in the intervein regions and eventually causing wing rupture. In most cases, this phenotype affected only one wing, and we did not observe it in driver control or *4xHand* > *UAS-Alk.EC* animals (Fig. 6A, quantification in Fig. 6A**´**). In addition to the dorsal vessel (Fig. 6B**, **DV), flying insects like *Drosophila* possess a pair of autonomous muscular pumps (so called wing hearts (WH), Fig. 6B**, **arrows) which ensure hemolymph circulation in the developing wing thereby contributing to wing unfolding and expansion [61, 62]. We wanted to further explore a potential connection between cardiac Alk signaling and wing heart morphogenesis using the *Alk*^*Tango*^ system as well as genetic manipulation of cardiac Alk signaling. Interestingly, analysis of the *Alk*^*Tango*^ > *LexAop2-CD4:tdTomato* reporter in a *HandC-GFP* background revealed Alk signaling activity in developing wing hearts when fibroblast-like precursor cells arranged into the sickle-shaped organ at ~ 40 h APF (Fig. 6C**)** [48]. At 80 h APF, we only detected faint *Alk*^*Tango*^ signals in mature wing hearts (Fig. 6D). We next used *4xHand* > driven *UAS-RedStinger* and *UAS-EYFP* to analyze wing heart morphology in animals with altered cardiac Alk signaling (Fig. 6E-H). In contrast to *4xHand* > control animals (Fig. 6E), cardiac overexpression of the Alk ligand Jeb led to a significant increase in wing heart muscle tissue (Fig. 6F) while Alk.EC had no significant effect on the size of these organs (Fig. 6G**,** quantification in Fig. 6H). In agreement with this, we observed strong hyperplastic growth of wing heart muscles upon cardiac overexpression of *UAS-Alk.Y1355S*, resembling the *Drosophila* ortholog of a point mutation found in aggressive human neuroblastoma (Fig. 6H, I). Interestingly, cardiac expression of a *UAS-Alk* construct did not increase wing heart size significantly (Fig. 6H, J) suggesting limited Jeb expression during wing heart development as well. Taken together, in addition to its role in sustaining cardiac function, Alk signaling affects growth of the accessory wing hearts.

## Discussion

In this study, we reveal a potential role for Alk signaling in the *Drosophila* heart. Cardiac expression of *Drosophila* Alk and its ligand Jelly belly has not been investigated previously although studies on zebrafish ALK family members [23], mouse [22]**,** and human [63] reported expression of ALK/LTK homologs and their ALKAL ligands in the heart, further suggesting a potential functional conservation for Alk signaling in this tissue. Exploration of the Mouse Gene Expression Database (GXD) identifies ALK receptor and ligand expression in RNA-seq datasets from cardiovascular tissues that support a role for this signaling axis in the heart [64]. Interestingly, a recent study in salamander looking at heart regeneration found the FAM150A (ALKAL1) ligand to be enriched in sub-epicardial endothelial cells during regeneration, raising the question of a role for ALK in cardiac homeostasis that could be tested in this model system [65].

In the fly [52], Alk expression is not observed during cardiac mesoderm specification and early heart formation in *Drosophila,* a process that is regulated by signaling from two other RTKs, FGFR/Heartless and EGFR [66]. However, single-cell profiling of stage 13–16 embryonic hearts recently revealed the presence of cardiac transcripts especially in svp-positive ostial cardioblasts [55] which is in line with our observations in late-stage embryos. Moreover, we detected cardiac Alk expression in larvae and adult flies accompanied by Jeb expression in larval and adult pericardial cells. *Alk* expression in cardiomyocytes of adult flies has been detected by single cell sequencing, whereas *jeb* transcripts were not detected in pericardial cells but in heart associated neurons [67]**.** While the *Drosophila* larval heart lacks innervation, the adult dorsal vessel is innervated by transverse nerves from bipolar neurons [68]. This might explain why cardiac Alk signaling at larval and pupal stages would require Jeb secretion from pericardial cells. Moreover, we observed *jeb*^*T2A*^ reporter expression in freshly emerged flies while tissue samples for single cell sequencing were generated from five-day old animals [67]. Therefore, it could be possible that Jeb expression is dynamic, changing from pericardial cells to heart innervating neurons in young flies. In addition, we cannot exclude the possibility that *jeb*^*T2A*^-positive dendritic branches of the ddaC neurons that are closely associated with the larval dorsal vessel could release Jeb by exocytosis [69]. Indeed, the role of *jeb* in ddaCs would be an interesting topic for future studies because these cells represent sensory neurons involved in nociception [70] and a recent study in mice connects ALKAL2 ligand expression in TRPV^+^ neurons to persistent pain [71].

In addition to Jeb and Alk expression, we also observed Alk^Tango^ activity in the larval and adult but not the embryonic heart further suggesting that Alk signaling is not required for heart formation but rather sustains cardiac function after hatching. Although information on ALK signaling in the human heart is sparse, one recent study reports an increase in cardiac arrythmias in patients treated with the ALK TKIs crizotinib and alectinib [72]. Moreover, an older study in mice reports cardiac hypertrophy, low blood pressure, and increased heart rate upon ubiquitous expression of a constitutively active form of the LTK receptor [73] that shares a high degree of structural and functional conservation with its closest relative ALK [74]. Because of the limited data, future comprehensive studies combining vertebrate models and genome-wide association studies (GWAS) will be required to address the function of human ALK signaling in cardiac function and disease.

Previous studies have identified important roles for EGFR, PDGF- and VEGF-receptor related (Pvr) as well as InR signaling for cardiac function in *Drosophila* [75–80]. In comparison to these studies, our work is the first to use a dimerization-sensitive reporter to reveal cardiac activity of a specific RTK during *Drosophila* development. In addition to this, our functional analysis uncovers an impact of ectopic activation of cardiac Alk on heart rate. One limitation of our study is the lack of observable defects on loss of Alk signaling, where further functional analysis is complicated because amorphic mutations in *Alk* and *jeb* cause lethality shortly after larval hatching [6]. Furthermore, CRISPR/Cas9-mediated, tissue-specific knock out of *Alk* and *jeb* is limited by the availability of suitable guide RNA-stocks and heart-specific drivers [77]. The lack of an observable cardiac defect upon Alk.EC misexpression and Alk-RNAi could indicate that Jeb/Alk-signaling in the heart is dispensable in a wild-type setting. Notably, Alk-signaling has been associated with brain sparing during nutrient restriction in flies [24] and ALK-deletion leads to increased energy consumption and reduced weight gain in obese mice [42]. It is therefore plausible to speculate that Alk-signaling could evoke cardiac responses in a diseased or physiologically challenged state. This idea is further supported by a recent assessment of ALK TKI-associated cardiotoxicity revealing that, in addition to the physiological burdens of cancer, old age and decreased cardiac function further increase the risk for drug-induced cardiac disorders [81].

As Alk likely shares downstream signaling pathways with other RTKs, it is difficult to clearly assign an observed effect on cardiac function to Alk signaling. However, ectopic expression of constitutively active EGFR in the heart has been reported to cause lethality at pupal stages [75] while manipulating cardiac Alk signaling had no such effect suggesting distinct roles of both RTKs on heart function. On the other hand, expression of either Pvr- [78] or Alk- (this study) transgenes in the heart exhibits similar effects on cardiac myofibrillar content and valve reporter gene expression suggesting that although these RTKs play a role in cardiac function, the results should be interpreted cautiously. Our results further reveal decreased average life span upon elevated cardiac Alk signaling as well as a rapid fitness decrease in young flies. This is interesting because a recent study links neuronal Alk signaling to aging and longevity [33] suggesting that in addition to a systemic role on lifespan, Alk signaling could also affect age-dependent organ physiology in a similar manner to insulin signaling [79]. Finally, our work also reveals an impact of Alk signaling on wing heart development in *Drosophila*. Previous analysis of *Hand*^*173*^ loss of function mutants revealed wing maturation defects identical to the phenotype observed after cardiac Jeb overexpression. In contrast to Jeb-induced wing heart hyperplasia, *Hand*^*173*^ wing hearts are reduced in size [82]; however, both conditions negatively affect wing heart organization suggesting that the observed wing phenotype is a result of impaired organ function. Although Alk signaling is essential for visceral but not cardiac *Hand* expression [60], it could be possible that Jeb-induced wing heart hyperplasia is a result of increased *Hand* expression. Intriguingly, blocking apoptosis restores *Hand*^*173*^-induced hypotrophy [82] which is in line with previous findings revealing *Hand* and its vertebrate homolog *Hand2* as suppressors of cardiac apoptosis [83, 84] as well as *Hand2* expression as downstream target of JNK signaling in zebrafish [85]. ALK signaling negatively affects apoptosis in human cells, and we recently revealed a link between Alk and JNK signaling in a *Drosophila* cell competition model [86, 87]. Therefore, it could be speculated that Jeb-associated wing heart growth is a result of blocked apoptosis mediated by an ALK > JNK > Hand signaling axis. On the other hand, it has been shown that ectopic activation of Ras/Raf/ERK and PI3K/Akt signaling pathways [84] leads to cardiac hypertrophy (CH) respectively increased organ size in mammalian models [88]. Moreover, hypertrophic cardiomyopathy (HCM) is a common feature of human RASopaties [89] while PI3KA regulates CH in diabetic cardiomyopathy [90]. Because Alk is a known upstream activator of Ras/Raf/ERK and PI3K/Akt in *Drosophila* and human cancers [91], signaling through these pathways could also be the cause of the observed wing heart phenotype. In addition, loss of function of Neurofibromatosis type I (NF1) and Shp2/PTPN11, two modulators of Alk associated Ras/Raf/ERK-signaling cause cardiac hypertrophy [92, 93]. In summary, our study revealed a potential role of Alk signaling for proper physiological function and growth of cardiac tissues. The evolutionary conservation of Alk expression in the human heart further suggests that ALK signaling should be considered in future studies on cardiac disease.

## Material and methods

### Key resource table

**Table:** 

**Reagent type (species) or resource**	**Designation**	**Source or reference**	**Identifiers **	**Additional information**
Genetic reagent *Drosophila melanogaster*	*Alk*^*TCS::LexA*^	This study		
Genetic reagent *Drosophila melanogaster*	*Alk*^*TEV*^	This study		
Genetic reagent *Drosophila* *melanogaster*	*Alk*^*mNeonGreenCT*^	This study		
Genetic reagent *Drosophila melanogaster*	*bap*^*HA*^	This study		
Genetic reagent *Drosophila melanogaster*	*Alk*^*1*^*/CyO, P{en1}wg*^*en11*^	[94]		
Genetic reagent *Drosophila melanogaster*	*P{13XLexAop2-mCD8::GFP}attP2*	Bloomington *Drosophila* stock center (BDSC)	BDSC: 32203	
Genetic reagent *Drosophila melanogaster*	*PBac{13xLexAop2-CD4-tdTom}VK00033*	Bloomington *Drosophila* stock center (BDSC)	BDSC: 77139	
Genetic reagent *Drosophila melanogaster*	*P{Hand-EGFP.C}*	[47]		
Genetic reagent *Drosophila melanogaster*	*TI{2A-GAL4}jeb*^*T2A-QF*^			Gift from Michael O´Connor
Genetic reagent *Drosophila melanogaster*	*P{4xHandC-Gal4}*	[95]		
Genetic reagent *Drosophila melanogaster*	*w*^*1118*^ *P{20XUAS-IVS-CsChrimson.mVenus}attP18; P{ppk-GAL4.1.9}2/CyO, P{2xTb1-RFP}CyO*	Bloomington *Drosophila* stock center (BDSC)	BDSC: 79598	
Genetic reagent *Drosophila melanogaster*	*PBac{10XQUAS-6XmCherry-HA}VK00018*	Bloomington *Drosophila* stock center (BDSC)	BDSC: 52269	
Genetic reagent *Drosophila melanogaster*	*P{UAS-RedStinger}*	Bloomington *Drosophila* stock center (BDSC)	BDSC: 8545, 8547	
Genetic reagent *Drosophila melanogaster*	*P{UAS-2xEYFP}AH3*	Bloomington *Drosophila* stock center (BDSC)	BDSC: 6660	
Genetic reagent *Drosophila melanogaster*	*P{Alk-GAL4.6.5}*	[52]		
Genetic reagent *Drosophila melanogaster*	*w*^*1118*^*; P{UAS-GMA}2/SM6a*	Bloomington *Drosophila* stock center (BDSC)	BDSC: 31775	
Genetic reagent *Drosophila melanogaster*	*P{Tl-cGFP}* also referred to as *toll*^*305*^-GFP	[96]		Gift from A. Paululat
Genetic reagent Drosophila melanogaster	*P{PTT-GA}Zasp66*^*ZCL0663*^	Bloomington *Drosophila* stock center (BDSC)	BDSC: 6824	
Genetic reagent Drosophila melanogaster	*P{UAS-Alk.EC.MYC}*	[27]		
Genetic reagent Drosophila melanogaster	*P{UAS-jeb.V}*	[60]		
Genetic reagent Drosophila melanogaster	*P{UAS-Alk}attP8*	This study		
Genetic reagent Drosophila melanogaster	*P{UAS-Alk.Y1355S}attP8*	This study		
Genetic reagent *Drosophila melanogaster*	*y*^*1*^ *M{vas-Cas9}ZH-2A w*^*1118*^*/FM7c*	Bloomington *Drosophila* stock center (BDSC)	BDSC: 51323	
Genetic reagent *Drosophila melanogaster*	*y*^*1*^ w^*^ *P{CaryIP}su(Hw)attP8*	Bloomington *Drosophila* stock center (BDSC)	BDSC: 32233	
Antibody	Anti-GFP (rabbit polyclonal)	Abcam	ab290	1:1000
Antibody	Anti-GFP (chicken IgY)	Abcam	ab13970	1:500
Antibody	Anti-lacZ (chicken IgY)	Abcam	ab9361	1:500
Antibody	Anti-Alk (rabbit polyclonal)	[52]		1:1000
Antibody	Anti-Alk (guinea pig polyclonal)	[52]		1:1000
Antibody	Anti-Jeb (guinea pig polyclonal)	[6]		1:1000
Antibody	Anti-RFP (Rabbit polyclonal)	Abcam	ab62341	1:2000
Antibody	Anti-mNeonGreen (Mouse monoclonal, 32F6)	Chromotek	32f6	1:250
Antibody	Anti-Mamo	[97]		1:100
Antibody	Anti-Cut homeobox (mouse monoclonal)	Developmental studies Hybridoma Bank (DSHB)	2B10	1:500
Antibody	Anti-EcR-B1 (mouse, monoclonal)	Developmental studies Hybridoma Bank (DSHB)	AD4.4	1:100
Antibody	Anti-Pericardin (mouse, monoclonal)	Developmental studies Hybridoma Bank (DSHB)	EC11	1:500
Antibody	Anti-Mef2 (Rabbit, polyclonal)	Developmental studies Hybridoma Bank (DSHB)		1:500
Antibody	Anti-Tinman (Rabbit, polyclonal)	Developmental studies Hybridoma Bank (DSHB)		1:750
Antibody	Anti-HA (mouse)	Biolegend	16B12	1: 500
Antibody	Anti-HA (Rabbit)	Cell signaling	C29F4	1:750
Other	DAPI	Thermo Fisher Scientific	D1306	1:1000
Other	FluoromuntG	SouthernBiotech	#0100–01	
Other	Alexa Fluor™ 647 Phalloidin	Thermo Fisher Scientific	A22287	1:1000
Antibody	Fluorophore coupled secondary antibodies	Jackson Immunoresearch ThermoFisher Invitrogen	706-166-148 706-606-148 111-166-144 111-546-144 111-606-144 703-546-155 715-166-151 715-546-151 A34055 32260 32230	1:1000 1:200 1:1000 1:1000 1:200 1:500 1:1000 1:1000 1:1000
Other	Animal sera for blocking	Jackson Immunoresearch	005-000-121 017-000-121	
Plasmid	pUASTattB	[98]		
Plasmid	pU6-BbsI-chiRNA	Addgene	45946	
Other	Halocarbon oil 700	Merck	9002-83-9	
Plasmid	pBlueScript-II-SK(-)-Alk.Bir*.HA donor	[99]		
Plasmid	pBlueScript-II-SK(-)-Alk^TCS::LexA^ donor pBlueScript-II-SK(-)-Alk^TEV^ donor pBlueScript-II-SK(-)-Alk^mNeonGreenCT^ donor pBlueScript-II-KS(-)-bap.3xHA donor	This study		
scRNA-seq data	GSE168774	[55]	*Drosophila* embryonic heart	cardiogenic progenitor population
Cell line	PC12, rat adrenal pheochromocytoma	PMID: 1065897		
Plasmid	pcDNA-dALK	[6]		
Plasmid	pcDNA-ALK-F1174L	PMID: 21838707		

#### *Drosophila* husbandry and fly genetics

Standard *Drosophila* husbandry procedures were followed. If not otherwise specified, we performed experiments and fly crosses at 25 °C, 60% humidity, under a 12 h: 12 h day and night cycle. Detailed information about *Drosophila* stocks and genetics is available on FlyBase (https://flybase.org/), the Database of *Drosophila* Genes & Genomes [100].

### Climbing assay

Flies of the indicated genotype were transferred into an empty plastic vial, marked at 4 cm height. The vial was sealed with a cellulose acetate plug. Flies were gently tapped to the bottom of the vial, and the time until 7 flies crossed the 4 cm line was measured with an iPhone timer. Each measurement was repeated two more times, and the resulting average time was used for statistical analysis in GraphPad Prism version 10.2.0.

### Developmental lethality and survival analysis

Flies were transferred to egg laying cages for 8 h. After hatching of first instar larvae, 50 larvae of the indicated genotype were transferred to food vials and the number of emerged flies was scored. For the survival assay, freshly hatched flies were transferred to food vials and kept at 29 °C. Scoring for dead individuals was done every day and vials were replaced every third day. Statistical analysis was performed using GraphPad Prism (version 10.2.0).

### Generation of *Alk*^*TCS::LexA*^, *Alk*^*TEV*^, and *Alk*^*mNeonGreenCT*^ using CRISPR/Cas9-mediated homology repair (HDR)

We modified the previously reported *Alk*^BirA*^ [99] donor construct by replacing the BirA* sequence with either *Drosophila* codon-optimized sequences (Genscript) encoding [1] TCS::LexA (4xGSAT linker, OLLAS-tag, two TEV cleavage sites, SV40 nuclear localizing sequence, LexA DNA binding domain, 3xVP16 minimal transactivation domain, and HA-tag), or (2) TEV (4xGSAT linker, HA-tag, TEV protease), or (3) mNeonGreen (Supplementary file 1) using NEBuilder® HiFi assembly (New England Biolabs) and standard cloning techniques. After sequencing (GATC services Eurofins), each donor was co-injected with the previously reported *pU6-BbsI-chiRNA Alk*-guide vector [99] into *y*^*1*^, *{Mvas-Cas9}ZH-2A, w*^*1118*^ embryos (BestGene Inc.). We identified successful HDR events by single fly PCR-screening and validated the desired genome modification by DNA sequencing (GATC services, Eurofins) in established isogenic stocks.

### Generation of *bap*^*HA*^ using CRISPR/Cas9-mediated HDR

We used CRISPR/Cas9-mediated genome editing to induce HDR in the *Drosophila bap* locus [57, 58]. Two CRISPR target sites (5'-AGCGGAGAGCGTTCACTCGG-3' and 5'-TGGGAGTGACCATGTCTCGG-3') were identified by the flycrispr optimal target finder tool [58] and corresponding single guide RNAs (sgRNAs) cloned into the pU6-BbsI-chiRNA gRNA expression vector (Addgene) as described in [58] to induce DNA double-strand breaks close to the *bap* stop codon. To achieve insertion of a 3xHA-tag construct, we provided a donor sequence to be co-injected together with the sgRNAs. The *bap.3xHA*, henceforth referred to as *bap*^*HA*^, donor DNA sequence was assembled by Integrated DNA Technologies (IDT) and contained homology arms (corresponding to 804 bp immediately upstream and 801 bp downstream of the *bap* stop codon) flanking a core sequence encoding three successive HA-tags (YPYDVPDYAYPYDVPDYAYPYDVPDYA). The donor DNA was cloned into pBluescript II KS (-) (GenScript) by using NEBuilder® HiFi assembly (New England Biolabs) and standard cloning techniques using flanking 30 bp sequences homologous to the pBluescript II KS (-) EcoRI-linearized vector. Both sgRNA and donor plasmids were injected into *y*^*1*^,*{Mvas-Cas9}ZH-2A,* *w*^*1118*^ embryos by BestGene Inc.. CRISPR candidate flies were balanced and screened for the *bap.HA* modification by PCR and subsequently verified by Sanger sequencing (GATC services, Eurofins).

### Generation of *UAS-Alk* and *UAS-Alk.Y1355S* lines

A codon optimized version of the *Alk* CDS (Genscript) was cloned into the pUASTattB vector [98] with EcoRI and XbaI restriction enzymes (New England Biolabs). In case of *UAS-Alk.Y1355S*, we used the NEBuilder® HiFi assembly kit (New England Biolabs) to introduce a point mutation (position 4063–4065 in the Alk CDS TAC > TCC) to alter the amino acid sequence accordingly. After sequencing, the constructs were injected into y1, w*, P{CaryIP}su(Hw)attP8 embryos (BestGene Inc.).

### Cell culture, treatment, lysis and immunoblotting

PC12 cells (ATCC CRL-1721) were cultured in RPMI 1640 medium supplemented with 7% heat inactivated horse serum and 3% non-heat inactivated fetal bovine serum (FBS) and a mixture of 1% penicillin/streptomycin under 37 °C, 95% humidity and 5% CO2 conditions. 2 × 10^6^ cells were electroporated in an Amaxa electroporator (Lonza, Basel, Switzerland), using 1.0 μg of empty pcDNA3 vector, pcDNA3-dAlk (*Drosophila* Alk) [6] or pcDNA3-ALK (F1174L) (human ALK) [101] construct as indicated with 100 μL of Ingenio electroporation solution (Mirus Bio LCC). Two days after transfection, cells transfected with either empty vector or pcDNA3-dAlk were treated with either 0.5 µg/ml of purified Jeb ligand (GenScript) or mock solution for 30 min. Cells were then harvested, and lysates were clarified by centrifugation at 14,000 rpm for 15 min at 4 °C. Samples were boiled in 1 × SDS sample buffer and analyzed by immunoblotting. Primary antibodies used for immunoblotting were anti-pALK (Y1278) rabbit mAb and anti-pERK1/2 (T202/Y204) rabbit mAb from Cell Signaling Technology; anti-β-Tubulin mouse mAb from ThermoScientific; anti-Alk antibody in house [52]. Horseradish-peroxidase-conjugated secondary antibodies goat anti-rabbit IgG and goat anti-mouse IgG were from Thermo Scientific.

### Neurite outgrowth assay

PC12 cells (2 × 10^6^) were electroporated with either 1.0 µg of empty pcDNA3 vector control, pcDNA3-dAlk (*Drosophila* Alk) [6] or pcDNA3-ALK-F1174L (human ALK) [101] positive control together with 0.5 µg pEGFPN1 as indicated by electroporation using Amaxa electroporator (Amaxa Biosystems) in 100 µl Ingenio electroporation solution (Mirus Bio LCC). After electroporation, cells were transferred to RPMI 1640 medium supplemented with 7% horse serum and 3% FBS and seeded into 24-well plates. Cells transfected with empty vector or pcDNA3-dAlk were cultured with either 0.5 µg/ml of purified Jeb ligand (GenScript) or mock solution. Two days after transfection, the percentage of GFP-positive and neurite-carrying cells versus GFP-positive cells was calculated under a Zeiss Axiovert 40 CFL microscope. To be judged as a neurite-carrying cell, the neurites of the cell had to reach at least twice the length of the cell body. Experiments were performed in triplicate and each sample within an experiment was assayed in duplicate.

### Microscopy

Samples were analyzed under a ZEISS Axio Imager.Z2 microscope. Images were acquired with ZEISS LSM800 or ZEISS LSM900 confocal microscopes with Plan-Apochromat objectives and ZEN Blue edition (versions 3.0 and 3.6) software.

### Fluorescence intensity measurement of cardiac collagen (Pericardin)

Segment A2 of 5-day old adult fly hearts was imaged by collecting Z-stacks. Control groups were imaged first to establish the laser intensity and exposure time for the entire experiment. The exposure time was based on image saturation (at a set point of approximately 70% of maximum saturation) to enable the comparison of fluorescence intensity across all genotypes. ImageJ (version 1.49) was used for processing.

### Actin disorganization analyses

Quantification of the disorganization of the cardiac myofibrils was performed with Voronoi’s Diagrams [102]. ImageJ software was used to outline the cardiac myofibrils and generate Voronoi’s areas. In well-organized hearts, the areas obtained were uniform with minimal variance. In contrast, in disorganized hearts, where circumferential fibers exhibited irregular spacing and convoluted paths, the generated areas varied significantly, resulting in a higher variance value.

### Wing heart area measurement

More than 100 animals per genotype were analyzed for wing heart phenotypes. For quantification, 12 pharate adult flies (~ 80 hpf) per genotype were removed from their pupal cases and mounted in Halocarbon oil 700. We used the Zeiss ZEN lite version 3.6 (blue edition) software to generate orthogonal projections from dorsal-view confocal stacks and to measure wing heart areas. To correlate wing heart area to overall body size (referred to as relative wing heart area) we divided the measured area by the product of the distance between the arches of the scutellar arms and the distance between the anterior margin and the posterior tip of the scutellum. GraphPad Prism version 9.5.1 was used for statistical analysis.

### Immunochemistry

Preparation and antibody staining of embryos, third instar larvae and adult flies was performed as described [103]**.** Embryos were dehydrated in an ascending ethanol series before clearing and mounting in methyl salicylate. Stained third instar larval and adult tissue dissections were embedded in FluoromountG.

### Video analysis of *Drosophila* pupal hearts

Heart videos from intact pupae (stage P4) were recorded in 1-min live sessions at a frame rate of 25 frames per second using a Zeiss Cell Discoverer 7 microscope equipped with an embedded Axiocam 712 camera system. Recorded movies underwent processing in ZEN Blue edition software (version 3.2). Subsequently, heartbeats per minute for each video were measured by manual counting over a 60 s period. Quantification was visualized with GraphPad Prism 10.2.0.

### Optical coherence tomography (OCT)

Cardiac function in adult *Drosophila* was measured using OCT. The system (Bioptigen) was built by as described by the Biophotonics Group, Duke University. Four-day-old flies were anesthetized by carbon dioxide (CO2) for 3–5 min and females were preselected from each group. Each fly was gently placed on a plate with petroleum jelly (Vaseline) for immobilization with the dorsal aspect facing the OCT microscopy source, then rested for at least 10 min to ensure the fly was fully awake. For each genotype, 10 flies were used. OCT was used to record the adult heart rhythm and heart wall movement at the same position, i.e., the cardiac chamber in the abdominal segment A2 of each fly. Each measurement was obtained at three different positions within the abdominal segment A2, these were averaged to obtain the cardiac diameter for that fly. M-mode images recorded the heart wall movement during the cardiac cycle. ImageJ software (version 1.49) was used to process the images. The diastolic dimension and systolic diameter were processed, measured, and determined based on three consecutive heartbeats. The heart period was determined by counting the total number of beats that occurred during a 15-s recording, then dividing 15 by the number of beats.

### Single-cell RNAseq analysis

The single-cell RNA sequencing dataset corresponding to the study "Single-cell profiling of the developing embryonic heart in Drosophila" [55], was retrieved from the publicly available repository under GEO accession number GSE168774. The dataset was reanalyzed using the Seurat package in R [104]. Cells from the cardiogenic progenitor population, which represent the heart cells in the overall population, were subsetted, and cellular heterogeneity was determined based on cell types described in the literature. The dataset was normalized using Seurat’s LogNormalize method, and the top 2000 highly variable genes were selected for downstream analysis. Data scaling and principal component analysis (PCA) were performed, with the first 30 principal components used to construct a uniform manifold approximation and projection (UMAP) for visualizing cellular heterogeneity. Expression levels of molecules associated with VM fusion downstream of Alk were visualized using stacked violin plots created with the scCustomize R package [105].

## Supplementary Information

Supplementary Material 1: Supplementary Figure S1. Recombinant Jeb ligand activates Alk receptor signaling in PC12 cells. Characterization of purified Jeb ligand. (A) Immunoblotting analysis of PC12 cells transfected with different constructs and treated with or without purified Jeb as indicated. pALK (Y1278) and pERK1/2 antibodies were used to indicate the activation of ALK and downstream signaling pathway. Alk antibody was used to detect ectopic expression of *Drosophila* Alk in PC12 cells. Cells transfected with pcDNA3-ALK-F1174L (human ALK) were used as positive control. Tubulin was used as loading control. (B) The percentage of transfected PC12 cells carrying neurites. Chart represents mean percentage ± SD from three independent experiments. (C) Representative light microscope images showing the neurite outgrowth of PC12 cells transfected and treated as indicated.

Supplementary Material 2: Supplementary Figure S2. Expression of *HandC-GFP*, Alk and *jebT2AQF*. (A-C) Antibody staining of *HandC-GFPDrosophila* embryos at stage 14 (A), 15 (B), and 16 (C). Anti-Alk appears in red, GFP (*HandC-GFP*) in green (D) Co-expression of *jeb*^T2A-QF^*>QUAS-mCherry *(red), *ppk1.9>UAS-CsChrimson.mVenus* (green), and anti-CT (blue) in larval ddaC neurons. Yellow encircled areas reveal the position of the heart in b/w images, arrows indicate Alk-positive cardioblasts. Scale bars are 20 µm.

Supplementary Material 3: Supplementary Figure S3. Expression of Bap^HA^ in Tin-positive pericardial cells. Antibody staining of Bap^HA^ embryos at stage 15. Bap^HA^ is primarily expressed in pericardial cells and co-localizes with Tinman markers (arrow)

Supplementary Material 4: Supplementary Figure S4. Heart-specific expression of either *Alk* or *jeb* RNAi does not affect heart structure, pericardin deposition or cardiac function in flies. (A) Adult (5-day-old females) heart phenotype induced by expression of either *Alk *or *jeb* RNAi. Cardiac actin myofibers were visualized by phalloidin staining (red). Pericardin was detected by immunofluorescence (green). Dotted lines delineate the outline of the heart tube. Scale bar = 40 µm. (B) Quantitation of adult heart Pericardin deposition relative to control. *n*=6 flies (5-day-old females) per genotype. (C) Images from *Drosophila* (4-day-old females) heartbeat videos obtained by optical coherence tomography (OCT). Representative images show changes in heart function induced by expression of either either *Alk* or *jeb* RNAi. (D) Quantitation of adult heart diastolic diameter. *n*=10 flies (4-day-old females) per genotype. (E) Quantitation of adult heart systolic diameter. *n*=10 flies (4-day-old females) per genotype. (F) Quantitation of heart period. *n*=10 flies (4-day-old females) per genotype (see A). Values are presented as mean along with the standard deviation (s.d). Statistical significance (*) was defined as ***P < 0.001 using Kruskal-Wallis H-test followed by a Dunn’s test.

Supplementary Material 5: Supplementary Movies M1-M3. Movie 1: Control conditions (*4xHand-Gal4 > UAS-RedStinger/+; UAS-EYFP/+*). Movie 2: in conditions of increased Alk signaling (*4xHand-Gal4 > UAS-RedStinger/UAS-jeb; UAS-EYFP/+*). Movie 3: in conditions of decreased Alk signaling (*4xHand-Gal4 > UAS-RedStinger/+; UAS-EYFP/UAS-Alk.EC*). All movies were recorded at 25fps for 30s, and heart measurements are shown in green channel.

## Data Availability

No datasets were generated during the current study. scRNA-seq data of the *Drosophila* embryonic heart (GSE168774) [55] was analysed in this study.
