# Supplementary figures and images for: AlkTango reveals a role for Jeb/Alk signaling in the Drosophila heart
Source: Cell Commun Signal. 2025 May 17;23:229. doi: 10.1186/s12964-025-02150-x (PMC12085853; doi:10.1186/s12964-025-02150-x)

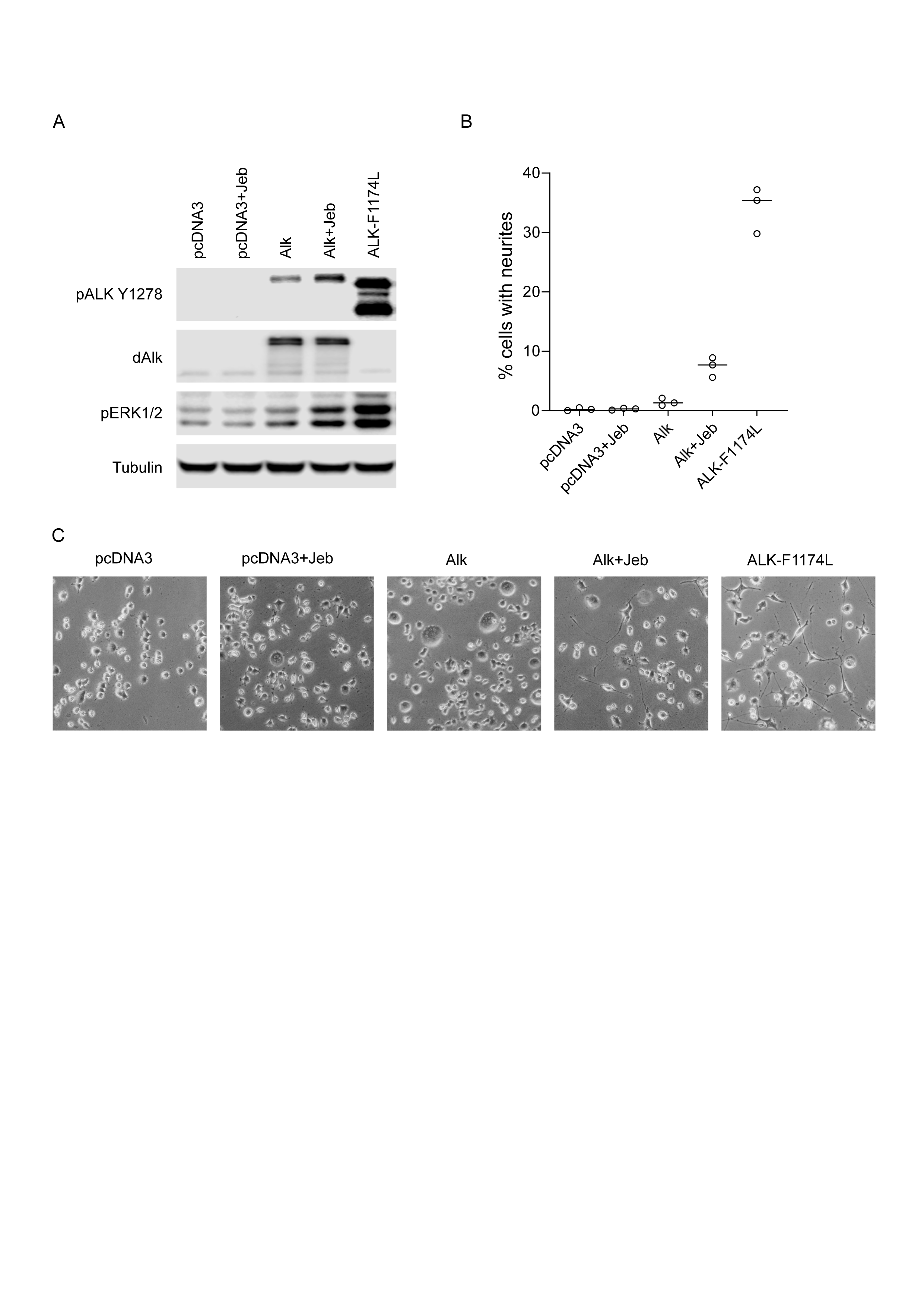

Supplement: Supplementary file 1 — Supplementary Material 1: Supplementary Figure S1. Recombinant Jeb ligand activates Alk receptor signaling in PC12 cells. Characterization of purified Jeb ligand. (A) Immunoblotting analysis of PC12 cells transfected with different constructs and treated with or without purified Jeb as indicated. pALK (Y1278) and pERK1/2 antibodies were used to indicate the activation of ALK and downstream signaling pathway. Alk antibody was used to detect ectopic expression of Drosophila Alk in PC12 cells. Cells transfected with pcDNA3-ALK-F1174L (human ALK) were used as positive control. Tubulin was used as loading control. (B) The percentage of transfected PC12 cells carrying neurites. Chart represents mean percentage ± SD from three independent experiments. (C) Representative light microscope images showing the neurite outgrowth of PC12 cells transfected and treated as indicated. [file 12964_2025_2150_MOESM1_ESM.zip › 12964_2025_2150_MOESM1_ESM.tif]

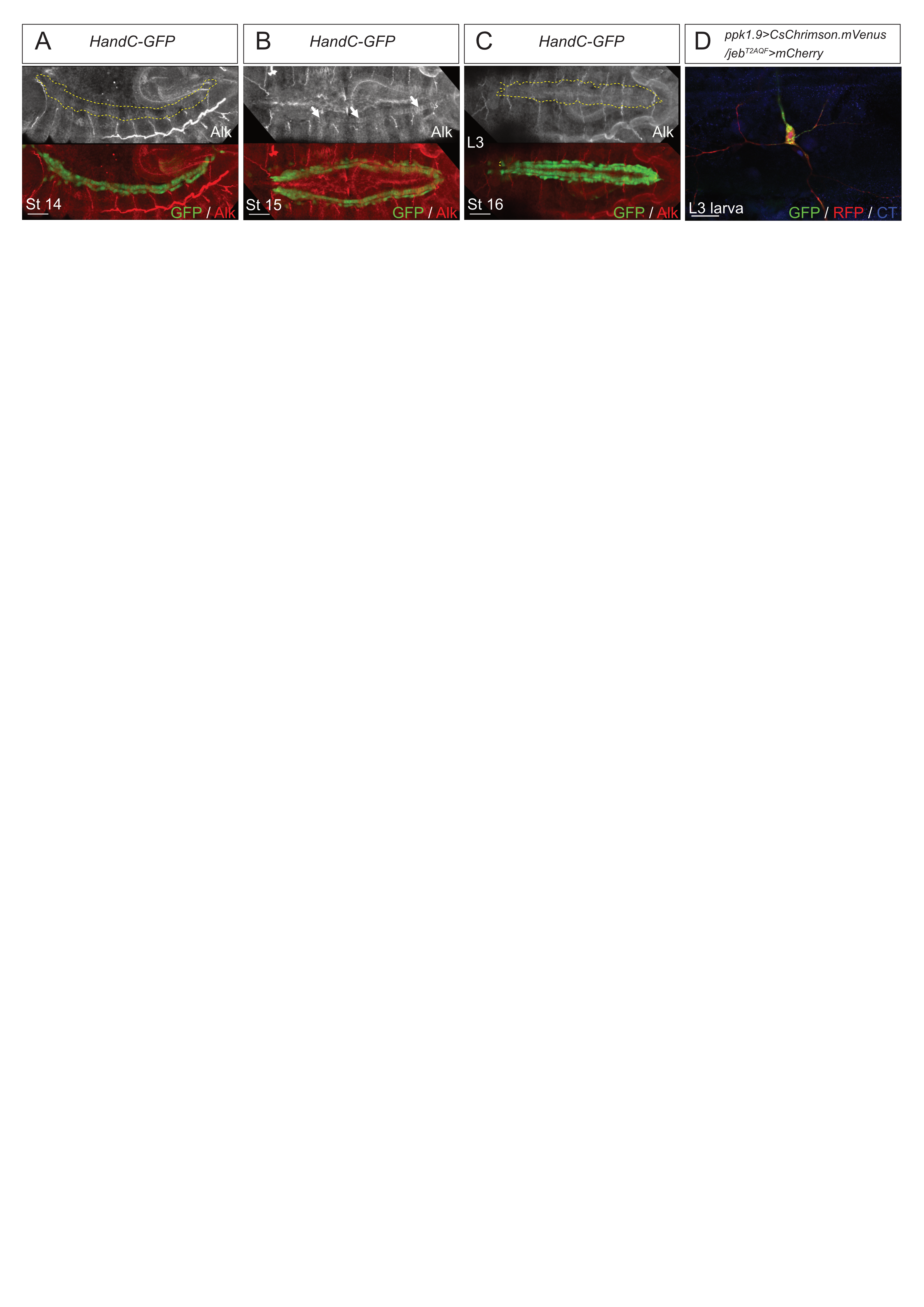

Supplement: Supplementary file 2 — Supplementary Material 2: Supplementary Figure S2. Expression of HandC-GFP, Alk and jebT2AQF. (A-C) Antibody staining of HandC-GFPDrosophila embryos at stage 14 (A), 15 (B), and 16 (C). Anti-Alk appears in red, GFP (HandC-GFP) in green (D) Co-expression of jebT2A-QF>QUAS-mCherry (red), ppk1.9>UAS-CsChrimson.mVenus (green), and anti-CT (blue) in larval ddaC neurons. Yellow encircled areas reveal the position of the heart in b/w images, arrows indicate Alk-positive cardioblasts. Scale bars are 20 µm. [file 12964_2025_2150_MOESM2_ESM.tiff]

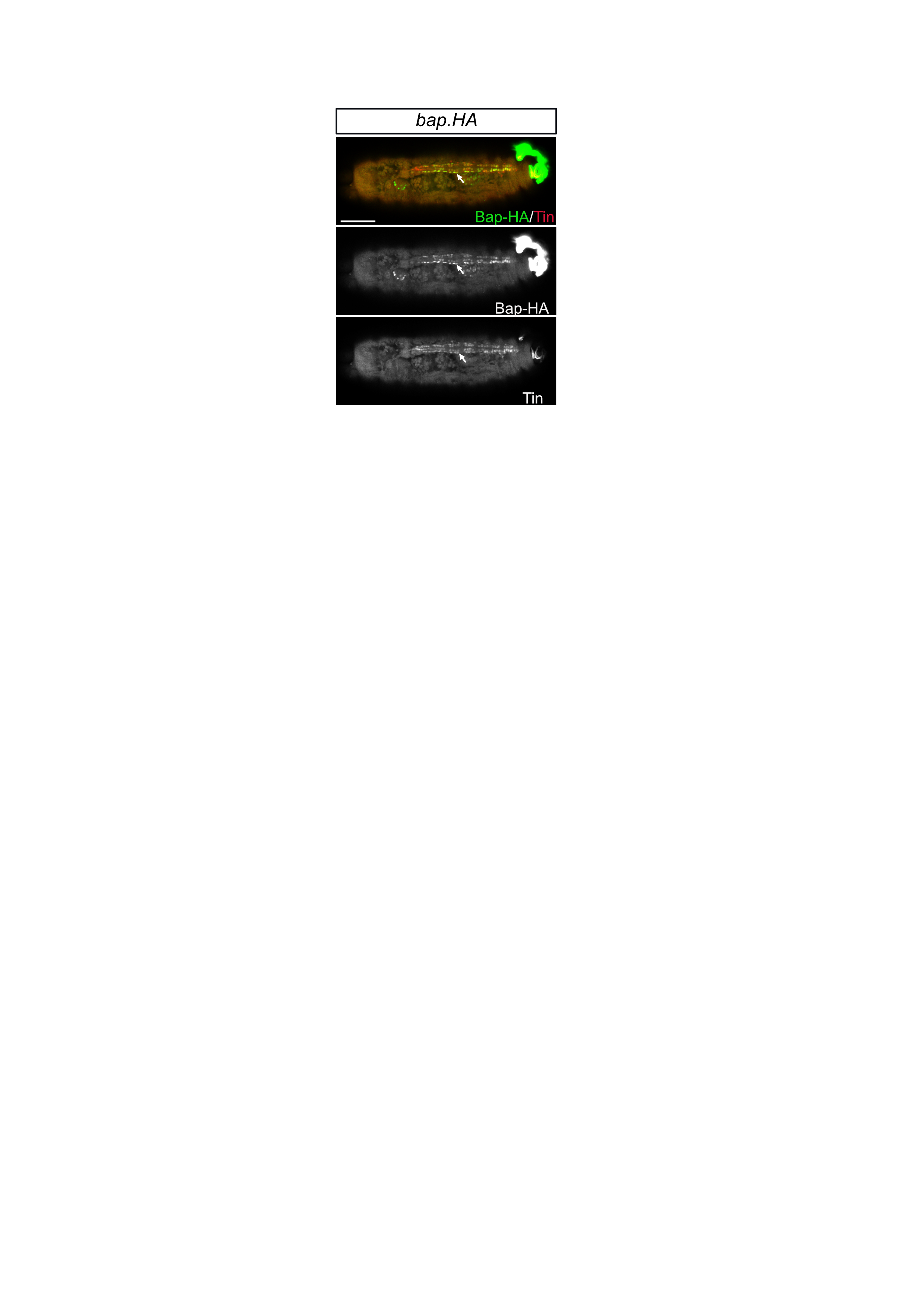

Supplement: Supplementary file 3 — Supplementary Material 3: Supplementary Figure S3. Expression of BapHA in Tin-positive pericardial cells. Antibody staining of BapHA embryos at stage 15. BapHA is primarily expressed in pericardial cells and co-localizes with Tinman markers (arrow) [file 12964_2025_2150_MOESM3_ESM.tiff]

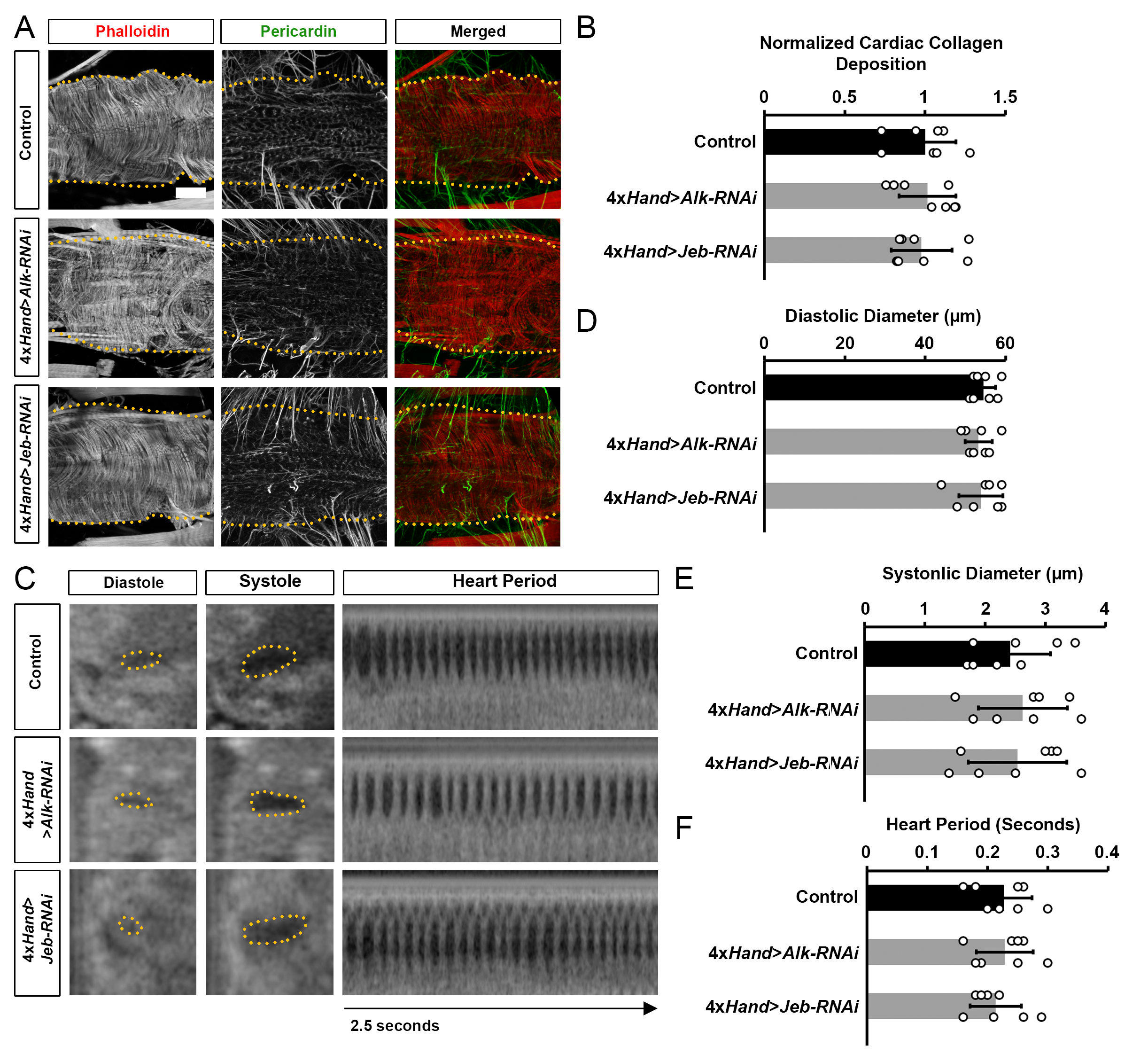

Supplement: Supplementary file 4 — Supplementary Material 4: Supplementary Figure S4. Heart-specific expression of either Alk or jeb RNAi does not affect heart structure, pericardin deposition or cardiac function in flies. (A) Adult (5-day-old females) heart phenotype induced by expression of either Alk or jeb RNAi. Cardiac actin myofibers were visualized by phalloidin staining (red). Pericardin was detected by immunofluorescence (green). Dotted lines delineate the outline of the heart tube. Scale bar = 40 µm. (B) Quantitation of adult heart Pericardin deposition relative to control. n=6 flies (5-day-old females) per genotype. (C) Images from Drosophila (4-day-old females) heartbeat videos obtained by optical coherence tomography (OCT). Representative images show changes in heart function induced by expression of either either Alk or jeb RNAi. (D) Quantitation of adult heart diastolic diameter. n=10 flies (4-day-old females) per genotype. (E) Quantitation of adult heart systolic diameter. n=10 flies (4-day-old females) per genotype. (F) Quantitation of heart period. n=10 flies (4-day-old females) per genotype (see A). Values are presented as mean along with the standard deviation (s.d). Statistical significance (*) was defined as ***P < 0.001 using Kruskal-Wallis H-test followed by a Dunn’s test. [file 12964_2025_2150_MOESM4_ESM.tif]
